# Moles and Mole Control on British Farms, Amenities and Gardens after Strychnine Withdrawal

**DOI:** 10.3390/ani6060039

**Published:** 2016-06-08

**Authors:** Sandra E. Baker, Stephen A. Ellwood, Paul J. Johnson, David W. Macdonald

**Affiliations:** The Wildlife Conservation Research Unit, Department of Zoology, The Recanati Kaplan Centre, University of Oxford, Tubney House, Abingdon Road, Tubney, Abingdon OX13 5QL, UK; stephen.ellwood@zoo.ox.ac.uk (S.A.E.); paul.johnson@zoo.ox.ac.uk (P.J.J.); david.macdonald@zoo.ox.ac.uk (D.W.M.)

**Keywords:** European mole, *Talpa europaea*, farms, amenities, gardens, mole control, strychnine, trapping, silage, questionnaire

## Abstract

**Simple Summary:**

Moles are burrowing mammals that are regarded as pests in Britain, and until 2006 they could legally be killed using strychnine poison. When strychnine was withdrawn there were fears that mole populations would increase. We surveyed farmers, amenity managers and householders about moles and mole control on their land in 2007, post strychnine withdrawal. Kill-trapping was by far the preferred control method used and control may be used more than can be justified by damage levels or the effect of control on damage. Mole traps are unregulated, unlike most other spring traps, and some might not meet current welfare standards. We found no evidence that mole activity had increased since a 1992 survey of farms.

**Abstract:**

Moles are considered pests in Britain, but this issue has been little studied. Lower welfare standards have been tolerated for moles than for most other managed wild mammal species, as use of both the controversial poison, strychnine, and unregulated traps have been permitted. Strychnine was withdrawn in 2006 and there were fears that mole populations would increase as a result. In 2007, we conducted a comprehensive, nationwide survey of land manager perceptions, opinions and behaviour regarding moles and mole control on farms, amenities and domestic gardens in Britain. We surveyed 2150 land managers (achieving a 59% response rate) and ground-truthed 29 responses. Moles were reported to be present on most farms and amenities, and 13% of gardens, and were more common in lighter soils. Where present, moles were usually considered pests, this being more likely in Wales, Scotland and northern England, on livestock and mixed farms, and on large, high-value amenities, e.g., racecourses and golf courses. Mole control followed similar patterns to mole presence. More control may occur than is economically, and therefore potentially ethically, justified. Control should be more carefully considered and, where necessary, more effectively targeted. Kill-trapping was the favoured recent and future method on farms and amenities, even if strychnine was to be reintroduced; however, because mole traps are currently unregulated, some might not meet current welfare standards if tested. We found no evidence for an increase in moles since a farm questionnaire survey conducted in 1992; this could have wider implications for future wildlife management policy changes.

## 1. Introduction

European moles (*Talpa europaea*) are solitary, fossorial insectivores. Each individual inhabits its own system of underground feeding tunnels, in which it hunts soil invertebrates, principally earthworms [1,2]. Moles are distributed across much of Europe and into Russia [3], and are widespread throughout Britain but do not occur in Ireland or on many of the British islands [1,2]. Moles produce conspicuous above-ground signs in the form of spoil heaps known as molehills. Mole tunnelling and molehill production are a cause of complaint among farmers, amenity managers and gardeners, and moles are widely considered pests [4].

Reported mole/molehill damage on farms includes pollution of silage, coverage of pasture, encouraging weed invasion and causing damage to plants, machinery and drainage systems/watercourses [4,5]. Damage reported on amenities and gardens is often merely aesthetic, but mole activity on racecourses, grass airstrips and sports fields may present risk of injury to people and animals [4,6]. Some aspects of farmers’ experiences and opinions of moles as pests, and of mole management conducted by farmers, have been studied in Britain [5] and Scotland [7], but no such research has been carried out with amenity managers or householders (domestic gardeners).

In a questionnaire survey, conducted in 1992, Atkinson *et al*. (1994) [5] found that 64% of farmers perceived moles as pests but that only 50% of survey respondents made efforts to manage them; the authors concluded that the damage attributed to moles was slight on most farms. No detailed data are available from the Scottish study [7]. The costs associated with mole damage and control are difficult to assess, but estimates for the total annual cost to farming in Britain range between £2.5 million in the late 1980s and up to £5 million in 1994 [2,4]. These figures equate to a range of £5.6–7.8 million at 2015 prices [8]. To put this in perspective, the annual cost of European badger (*Meles meles*) damage caused to crops, or through burrowing or predation, in England and Wales was estimated to be somewhere between £21.5–41.5 million per annum in 1997 [9], equating to £35–68 million in 2015.

Various methods are used for managing moles and molehills in Britain, including kill-trapping (spring-trapping), gassing with phosphine, live-trapping, shooting and ultra-sonic deterrents, as well as harrowing and rolling to flatten molehills. No poisons or chemical repellents are currently approved for use with moles. Renardine^TM^, a bone oil-based repellent [10], was approved until 2005, when the license was withdrawn across Europe following concerns over the transmission of Bovine Spongiform Encephalopathy (BSE) [11]. Strychnine is an alkaloid poison. Acting through competitive inhibition of glycine (a neurotransmitter), strychnine allows unimpeded stimulation of motor neurons thus causing the striated muscles to produce generalised rigidity and tetanic seizures [12]. Ultimately this leads to death through exhaustion or through asphyxia arising from paralysis of the respiratory muscles. Strychnine has been described as dangerous and inhumane [5] and was banned for wildlife management under the Animals (Cruel poisons) Regulations of 1963, and yet its continued use was allowed only with moles. Atkinson *et al*. [5] suggested this exemption was tolerated because: (1) mole damage was perceived to demand action; (2) strychnine was the only poison cost-effective on an agricultural scale (and no suitable alternative was available); (3) moles died underground where their painful deaths could not be seen.

For a pesticide such as strychnine to be used legally, not only must the pesticide not be illegal for the particular purpose, but the pesticide must also be approved for that use (now by the Chemicals Regulation Directorate [13]. By the early 1990s, the number of permits for strychnine use was being reduced [5], and in 2006 strychnine approval for use with moles was withdrawn, as part of an EU pesticide review, when manufacturers failed to produce the necessary health and safety data [14]. This followed years of concern about the humaneness of strychnine poisoning, the potential for its misuse, and the significant costs involved in administering and policing its use [4,5,15]. Strychnine also presented the potential threat of accidental primary or secondary non-target poisoning of wildlife and companion animals (see [15,16]). However, strychnine was considered to be the most cost-effective method for managing moles on a large scale, although kill-trapping by skilled operators may be better for removing small numbers of moles [4,5]. Strychnine is reported to have once been the main method of managing moles on British farms [7]; see also [4,5]. It has been suggested that, because of the relatively low cost of strychnine treatment, more moles may have been killed than could have been justified [4]. And it is possible that, if these had not been poisoned, some may have died anyway as part of the naturally “doomed surplus” (see Errington [17]).

Although strychnine use in mole control was already declining before it was banned, in 2004/2005 sufficient strychnine was sold in Britain to kill almost 800,000 moles [1] and 3000 users were licensed [14]. It was predicted that the loss of strychnine would force users to reconsider the costs and benefits of managing moles using alternative methods, and that trapping would become the primary method of mole management, while for some managers, taking no action may become the best choice [4,5]. Because of concern that no suitable alternative mole management method was available, it was suggested that mole populations would increase [4,18], and it was subsequently claimed that they had [19,20,21]. Although strychnine approval was withdrawn in 2006, the legal exemption to use it for mole control remains, so if strychnine was to be re-approved in future, it would become legal to use it again.

We conducted a comprehensive, nationwide questionnaire survey to examine land manager perceptions, opinions and behaviour regarding moles and mole control on farms, amenities and domestic gardens throughout Britain, and factors affecting these. We set out to compare some farmer responses with those obtained in Atkinson *et al*.’s 1992 survey [5]. We also aimed to estimate the impact, if any, of strychnine withdrawal on mole management. We conducted field surveys to ground-truth a sample of questionnaire respondents for validation purposes. We found that where moles were present, they were commonly regarded as pests, and often controlled. Our results also suggest that more control may be conducted than is necessary and that it may be poorly targeted. To our knowledge, this is the first published survey of mole management issues on amenities and domestic gardens, and the largest survey conducted to date regarding moles on farmland.

## 2. Materials and Methods

This study was granted ethical approval by the Central University Research Ethics Committee at the University of Oxford (approval reference was SSD/CUREC1/07-003).

### 2.1. Questionnaire Survey

We designed a postal survey using Dillman’s Tailored Design Method [22] to optimise survey response rate, including multiple contacts with potential respondents: (a) pre-notice letter; (b) questionnaire and covering letter; (c) thank you/reminder postcard; (d) replacement questionnaire to non-responders. We obtained endorsement for the survey from: The National Farmers’ Union Wales, The National Farmers’ Union of Scotland, The British Association for Shooting and Conservation, The Game Conservancy Trust (now the Game and Wildlife Conservation Trust), The National Trust, The Royal Horticultural Society, The Racecourse Association, The Airport Operators’ Association, and The British and International Golf Greenkeepers’ Association. The support of these organisations was mentioned in the covering letter accompanying questionnaire mailings.

We devised separate questionnaires for farmers, amenity managers and householders, wherever possible using similar-style questions across the three target groups to allow comparison of responses. Questions covered: (a) Your farm/amenity/garden or allotment; (b) Moles on your farm/amenity/garden or allotment; (c) Mole control on your farm/amenity/garden or allotment; (d) Your opinions about mole control; (e) Strychnine poison; (f) Interest in participating in a follow-up study (ground-truthing). Questionnaires were marked with individual identity numbers and printed as A4 double-sided booklets. Questionnaires were piloted, and contacts and questionnaires were translated from English into Welsh (recipients in Wales received versions in both languages). We used a system detailed under “Recipient database abstraction”, in Text S1, to randomly select 1204 farmers, 551 amenity managers and 504 householders from across England, Scotland and Wales as survey recipients. Contacts were posted to the 2259 recipients as follows: (1) 21 June 2007 pre-notice letter (all recipients); (2) 28 June 2007 questionnaire and covering letter (all recipients); (3) 4 July 2007 thank you/reminder postcard (all recipients); (4) 18 July 2007 replacement questionnaire (non-responders only). Full details of the questionnaire survey method are given in Text S1 and examples of the contacts and of the questionnaire are given in Text S2–S6.

### 2.2. Questionnaire Analysis

Questionnaire data were stored in a Microsoft Access database and analysed using SAS statistical software (The SAS Institute, Cary, NC, USA) [23]. We used χ^2^ and (where expected counts were low) Fisher’s Exact tests to examine relationships among categorical variables in contingency tables. We used Friedman Tests to analyse ranked monthly mole activity and ranked features of a mole control method. Kruskal-Wallis χ^2^ tests were used to compare costs data across categories of respondent type.

### 2.3. Ground-Truthing Survey

We ground-truthed a proportion of questionnaire respondents, to assess the accuracy of their reports regarding the presence of moles and the scale of mole activity on their land. Ground-truthing took place in spring and early summer 2008 and 2009 to coincide with periods of peak mole activity and control as reported in our questionnaire study, and by Atkinson *et al.* [5]. We selected potential participants from questionnaire respondents who indicated an interest in participating in follow-up work. We attempted to identify a representative sample of 29 questionnaire respondents, consisting of 17 farmers (59%), eight amenity managers (28%) and four householders (14%), closely reflecting the proportions in which they responded to the questionnaire (57%, 24%, and 19%). The farms included seven mixed, six livestock, two arable, and two other farms, and the amenities were three ornamental gardens, two golf courses, one racecourse, one ancient monument and one park.

Ground-truthing was a two-stage process consisting of a 30–60 min semi-structured face-to-face interview, followed by a field survey. Interviews were intended largely to help us identify suitable sites for ground-truthing using field survey. During the interview participants were asked to identify, on maps and aerial photographs, six sites (fields/areas), on their land, which had at that time the greatest (three sites) and least (three sites) mole activity. Where a respondent reported no current mole activity on his land, he was asked to nominate just three “no activity”sites. We recorded further information for each site including current land-use (e.g., pasture, cereal, mown grass) and site access points. Following each interview, we selected two sites for survey—one each identified by the participant as having high mole activity and low mole activity; we attempted to balance the representation of different field/area types, then prioritising sites with the most and the least reported mole activity. Where a participant nominated only “no activity” sites we selected one for survey. Of the 29 participants, 23 reported both high and low activity sites and six (one amenity manager, one farmer and four householders) reported no current mole activity; so, altogether we surveyed 52 sites. Field surveys were conducted within a few days of the interview. At each site we implemented a simple survey strategy that could be adapted to different field circumstances; this involved conducting approximately 30 full-point surveys at each site to record molehill activity. Full details of the ground-truthing survey method are given in Text S7.

During the ground-truthing interview we also asked participants whether they felt that mole activity had increased, decreased or stayed the same on their land over the previous 5 years.

### 2.4. Ground-Truthing Analysis

First we checked whether land managers had successfully predicted molehill presence and/or absence on their land. Then, using our survey data, we calculated molehill density per site and compared these estimates with land managers’ reported perception of relative molehill activity on their nominated sites. Further details of data manipulation and analyses are given in Text S8.

## 3. Results

### 3.1. Questionnaire Response Rates

We included responses received up to the 11 November 2007. After excluding ineligible recipients, there were 2150 potential respondents comprising 1143 farmers, 526 amenity managers and 481 householders. We achieved an overall response rate of 59% (*n* = 1265), with response rates differing among respondent types (farmers 63% (*n* = 720), amenity managers 58% (*n* = 303), householders 50% (*n* = 242) (χ^2^_(2)_ = 22.9, *p* < 0.001, Table S1)), and regions, with more in Scotland and in the north and south-west of England (χ^2^_(4)_ = 12.3, *p* = 0.015, Figure S1). There was no evidence that respondent type varied with region (χ^2^_(8)_ = 6.7, *p* = 0.574). More information on questionnaire response rates is available in Text S9.

### 3.2. Questionnaire Responses

#### 3.2.1. Factors Affecting Mole Presence and Pest Status

We examined factors affecting reported historic mole presence (whether they had ever had moles on their land) and mole pest status. Where “mole presence” and “pest status” are used here we refer respectively to mole presence at any time in the past and perceived mole pest status. In the questionnaires, respondents were asked first whether moles were present on their land and then whether they considered moles to be pests. There is at least some possibility that asking whether a particular species is a pest might “lead” some respondents to say that it is when that species might not have appeared on an unprompted list of pests. However, this approach is transparent and avoids bias when making comparisons across categories of, for example, respondent type, farm enterprise type *etc*.

**Figure 1:**
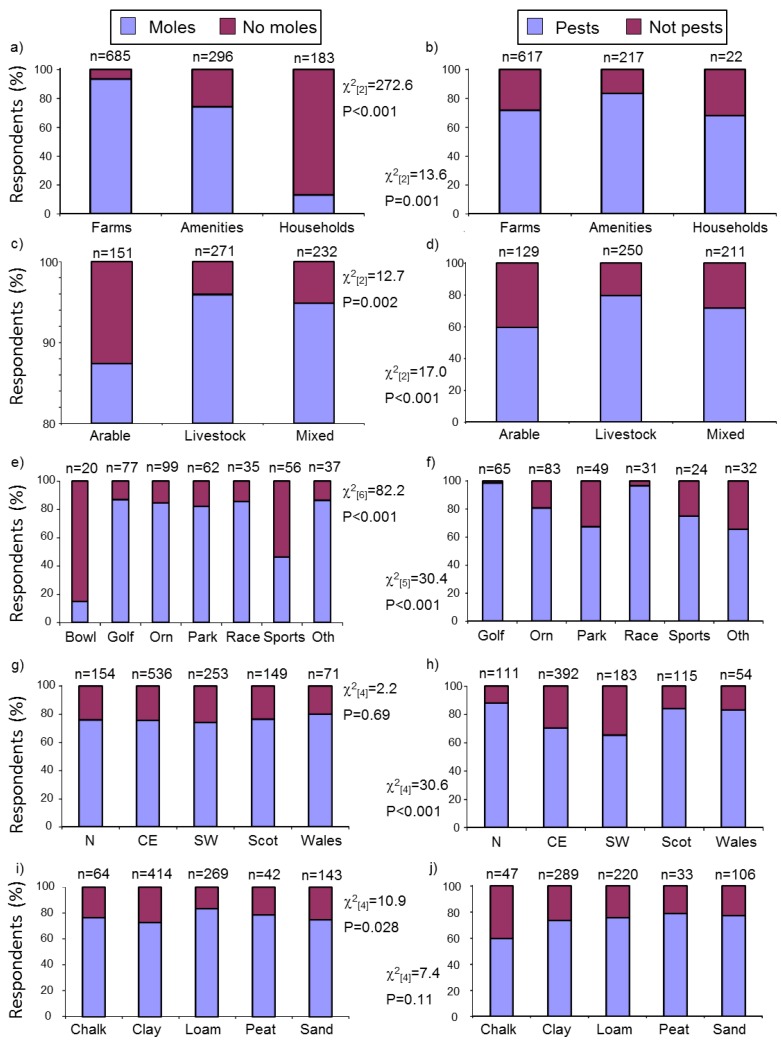
Mole presence and mole pest status, respectively, by: (**a**, **b**) respondent type; (**c**, **d**) farm enterprise type; (**e**, **f**) amenity type, (**g**, **h**) region; (**i**, **j**) soil type. Amenity types are: Bowl = bowling greens, Golf = golf courses, Orn = ornamental gardens, Park = parks, Race = racecourses, Sports = sports fields and Oth = other amenities. Regions are: N = northern England, CE = central and eastern England, SW = south-western England, Scot = Scotland and Wales = Wales. Statistics shown are results of χ^2^ tests.

Reported mole presence differed significantly among respondent types with most farmers and amenities, but only 13% of householders, reporting that they had ever had moles on their land (Figure 1a). The majority of respondents reporting mole presence considered them to be pests and the likelihood of them doing so differed significantly among respondent types, with amenity managers the most likely to consider moles on their land as pests (Figure 1b). Reported mole presence and pest status were related to both farm enterprise and amenity type (Figure 1c–f). Livestock farms were most likely to report mole presence followed by mixed and then arable farmers, and of these farmers with moles, livestock farmers were also most likely to consider them pests (Figure 1c,d). Mole presence was reported by a minority of bowling greens and sports fields, but by most golf courses, ornamental gardens, parks, racecourses and other amenities (Figure 1e). Of those with moles, over 96% of golf courses and racecourses considered moles pests together with the majority of other amenities (bowling greens were excluded because so few reported mole presence) (Figure 1f).

There was no evidence that mole presence varied regionally (Figure 1g), although respondents from Wales, Scotland and northern England were more likely to consider moles on their land as pests (Figure 1h). Soil type was related to mole presence, with moles occurring more frequently on loamy soils and least on clay (Figure 1i), but where moles were present there was no effect of soil type on pest status (Figure 1j). Soil type also varied with region (χ^2^_(24)_ = 93.5, *p* < 0.001), with chalky and clay soils more prevalent in central and eastern, and south-western, England and loamy-peaty-sandy soils more common in Scotland, Wales and northern England (Figure S2), thus suggesting that regional variation in the presence of moles could be at least partly related to soil type.

#### 3.2.2. Factors Affecting Recent Mole Activity and Damage

We examined the impact of the same factors on recent mole activity and whether respondents considered this activity to be damaging. Where “recent mole activity” and “damage” are used here we refer respectively to mole activity in the previous 12 months and perceived mole damage. Data on recent activity indicated that respondents from farms (particularly livestock and mixed farms) and amenities (particularly golf courses), and from Wales and northern England, reported a greater incidence of recent activity than did householders and respondents from Scotland, central and eastern England and south-western England (Figure S3). When those respondents reporting recent mole activity were asked if they considered this to constitute damage, amenity managers, livestock farmers and respondents from Wales and northern England, were more likely than others to say that they did. More details are available in Text S10.

#### 3.2.3. Factors Affecting Mole Control

We examined factors affecting whether historic and recent mole control had been conducted. Where “historic control” and “recent control” are used here we refer, respectively, to control conducted at any time in the past and control conducted in the previous 12 months.

Historic control was affected by respondent type, farm enterprise, amenity type, region and soil type (Figure S4a,c,e,g,i). Patterns of control were generally similar to those reported for mole presence but occurring at a lower level (Figure S4a–j, e.g., while 93% of farmers reported mole presence, 65% reported control). One key difference, however, was that historic mole control was related to region (whereas historic presence was not), with control more prevalent in Wales, Scotland and northern England (χ^2^_(4)_ = 33.1, *p* < 0.001, Figure S4g).

Of those respondents that had ever controlled moles, there was no evidence for difference among farm enterprises, amenity types or regions, in the proportion that controlled them recently (Figure S4d,f,h). This suggests that whether a historic controller tended to control moles from year to year was not affected by these factors. However the likelihood that a historic mole controller controlled moles recently was affected by respondent type and soil type, with amenity managers and farmers, and respondents on peaty or sandy soil, being more likely to have controlled moles recently and perhaps therefore regularly (Figure S4b,j).

#### 3.2.4. Costs of Mole Damage and Control

Mean costs of damage and control were calculated for respondents reporting damage or control respectively in the previous year. Total control costs comprised professional costs (as reported) and non-professional costs (which were calculated by adding the reported cost of materials to the estimated cost of labour—produced by multiplying the reported number of hours spent on mole control by the UK hourly minimum wage rate for people aged 22 years or above (£5.73, the equivalent of £7.10 in 2014 [8])).

**Figure 2:**
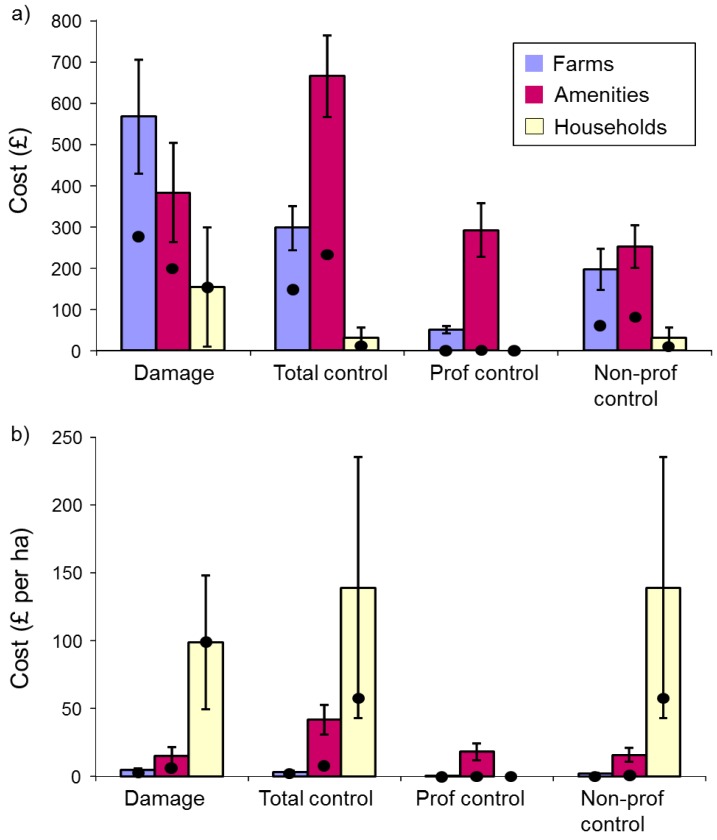
Damage and control costs for farms, amenities and households (respondents with damage or conducting control): (**a**) Raw costs per holding (damage-farms *n* = 26, amenities *n* = 21, households *n* = 2; total control-farms *n* = 157, amenities *n* = 98, households *n* = 5; professional control-farms *n* = 269, amenities *n* = 130, households *n* = 6; non-professional control-farms *n* = 168, amenities *n* = 108, households *n* = 5); (**b**) Costs·ha^−1^ (damage-farms *n* = 24, amenities *n* = 19, households *n* = 2; total control-farms *n* = 150, amenities *n* = 89, households *n* = 4; professional control-farms *n* = 255, amenities *n* = 119, households *n* = 5; non-professional control-farms *n* = 161, amenities *n* = 99, households *n* = 4). Bars are means with standard errors, and dots indicate median values.

There was no evidence that raw damage costs per holding differed among respondent types (Kruskal Wallis (KW) χ^2^_(2)_ = 1.58, *p* = 0.45). In contrast, raw total control costs differed significantly among respondent types (Figure 2a, KW χ^2^_(2)_ = 21.70, *p* < 0.001), with those for amenities being greatest, followed by farms and then households; underlying professional and non-professional control costs followed a similar pattern but did not differ significantly among respondent types (KW χ^2^_(2)_ ≤ 5.56, *p* ≥ 0.06). When costs were expressed per ha, damage, total control and professional control costs all differed significantly among respondent types, with those for households being greatest, followed by amenities and then farms (Figure 2b, KW χ^2^_(2)_ ≥ 5.82, *p* ≤ 0.05), but professional control costs did not (KW χ^2^_(2)_ = 4.71, *p* = 0.10).

More respondents reported control costs than reported damage costs (see sample sizes in Figure 2b legend: farms 150/24, amenities 89/19, householders 4/2), and amenities spent more on control than they sustained in damage, while farmers and a very small sample of householders sustained a greater value of damage than they spent on control (based on median values).

**Figure 3:**
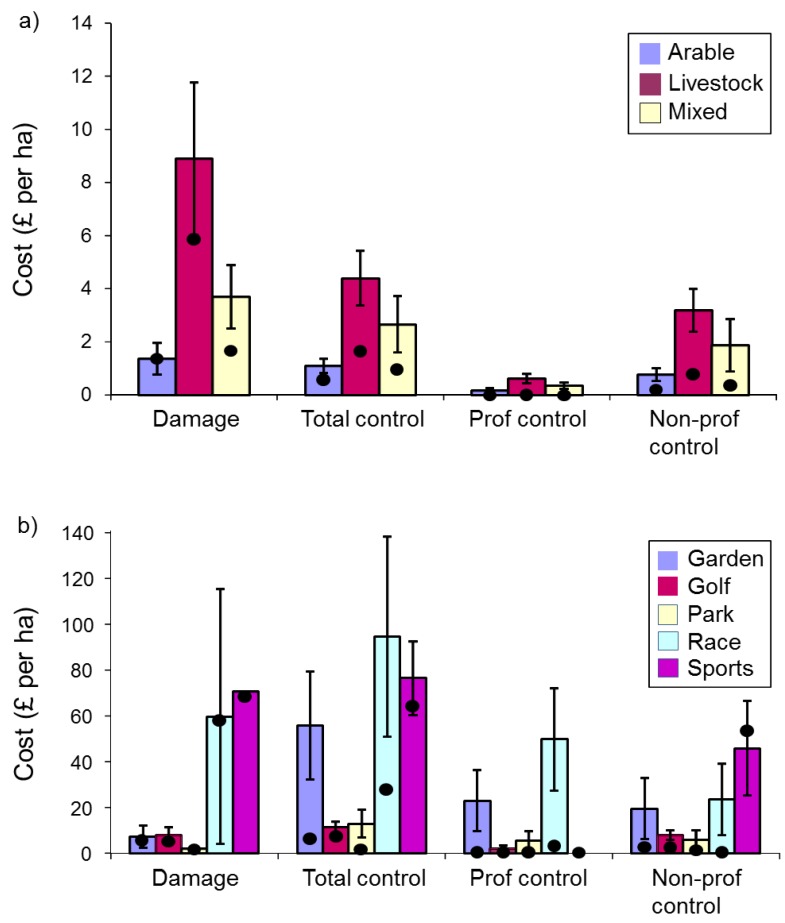
Damage and control costs·ha^−1^, and total financial losses due to mole activity·ha^−1^, by: (**a**) farm enterprise (damage-arable *n* = 5, livestock *n* = 7, mixed *n* = 11; total control-arable *n* = 28, livestock *n* = 73, mixed *n* = 42; professional control-arable *n* = 48, livestock *n* = 120, mixed *n* = 78; non-professional control-arable *n* = 30, livestock *n* = 78, mixed *n* = 45); (**b**) amenity type (damage-gardens *n* = 3, golf courses *n* = 7, parks *n* = 6, racecourses *n* = 2, sports fields *n* = 1; total control-gardens *n* = 28, golf courses *n* = 27, parks *n* = 14, racecourses *n* = 15, sports fields *n* = 3; professional control-gardens *n* = 44, golf courses *n* = 35, parks *n* = 16, racecourses *n* = 19, sports fields *n* = 3; non-professional control-gardens *n* = 28, golf courses *n* = 29, parks *n* = 15, racecourses *n* = 20, sports fields *n* = 5). Bars are means with standard errors, and dots indicate median values.

Damage and control costs·ha^−1^ for farmers reporting damage or control, respectively, were greatest for livestock farmers, followed by mixed and arable farmers. Damage costs and total control costs varied marginally significantly (KW χ^2^_(2)_ = 5.54, *p* = 0.063) and significantly (KW χ^2^_(2)_ = 15.07, *p* < 0.001), respectively, among farm enterprises, but professional and non-professional control costs did not (Figure 3a, KW χ^2^_(2)_ ≤ 2.69, *p* ≥ 0.260).

Sample sizes were small for some amenity types but nevertheless there was a significant difference among amenity types in both total and professional control costs·ha^−1^ (KW χ^2^_(4)_ ≥ 11.5, *p* ≤ 0.02), with racecourses suffering the greatest total costs·ha^−1^ (NB the relatively large costs for sports fields were reported by very few respondents). There was no significant difference in damage costs or non-professional control costs (Figure 3b, KW χ^2^_(4)_ ≤ 7.03, *p* ≥ 0.13).

#### 3.2.5. Seasonal Patterns in Mole Activity and Control

**Figure 4:**
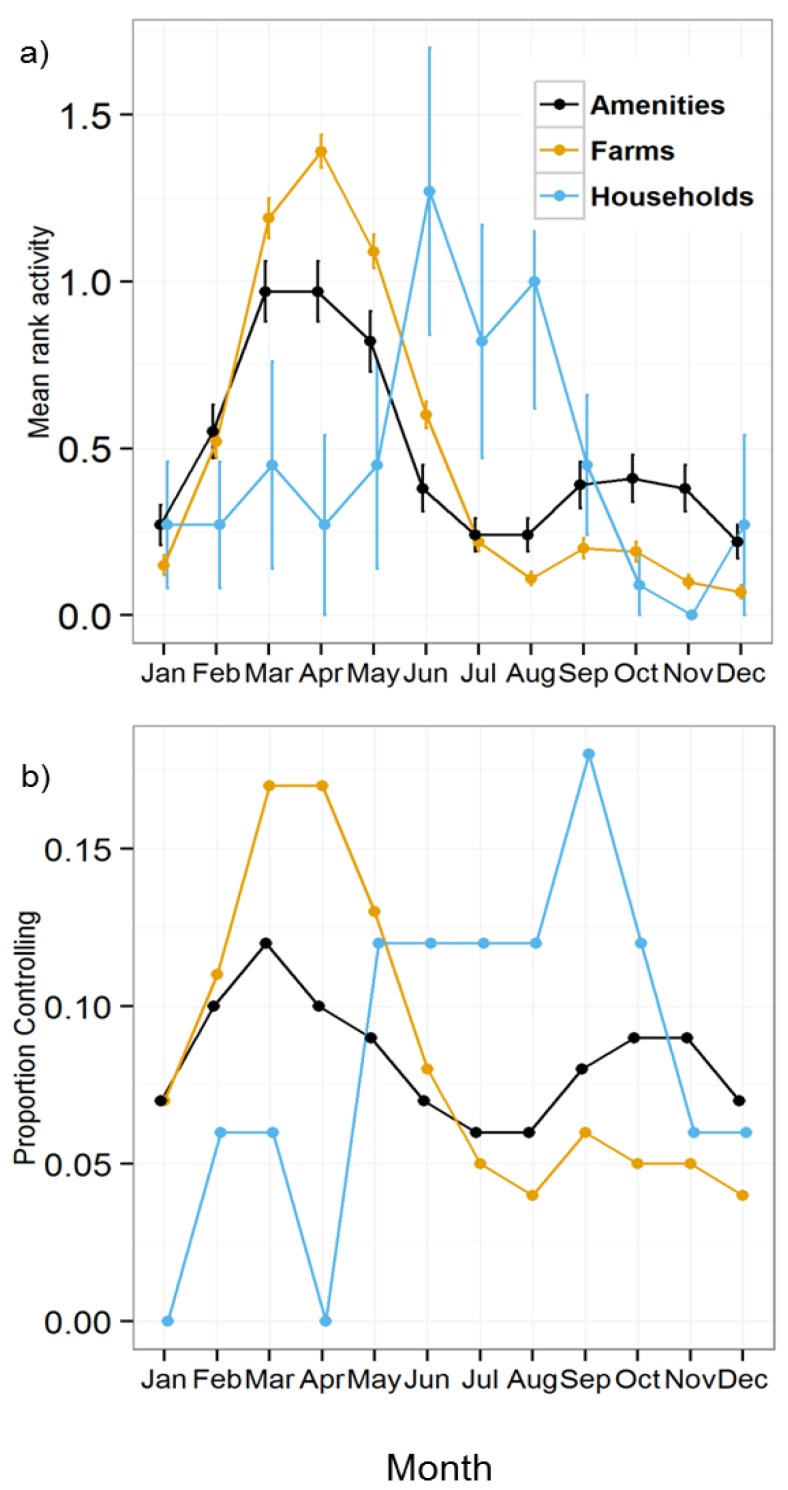
Seasonal patterns in mole activity and control in the previous year, by respondent type, based on: (**a**) mean ranked monthly activity (farms *n* = 496; amenities *n* = 168; households *n* = 11), standard errors are shown; (**b**) proportion of respondents conducting control each month in the previous year, by respondent type (farms *n* = 272; amenities *n* = 132; households *n* = 6).

We examined seasonal patterns in recent mole activity and control. First we asked respondents to rank the three months in the previous year in which mole activity was greatest. We then allocated a “3” to the month ranked highest, “2” to the month ranked second and “1” to the month ranked third. From these numbers we produced mean relative rankings for monthly activity; overall, activity reported by all respondents varied across the months (Friedman’s χ^2^_(11)_ = 1331.0, *p* < 0.001). Patterns on farms and amenities were broadly similar with a larger spring peak and a smaller one in the autumn, with the spring peak being more exaggerated for farmers than amenities and the autumn peak more pronounced for amenities than farmers (Figure 4a). The very small number of householders that responded to the question reported a single peak in activity in the summer.

We asked respondents to report the months in which they conducted mole control during the previous year and plotted the proportion of responses for each month (Figure 4b). Control patterns broadly followed activity patterns. Overall, control reported by all respondents varied over the year (χ^2^_(11)_ = 259.7, *p* < 0.001). Patterns in control differed between farmers and amenities (χ^2^_(11)_ = 67.0, *p* < 0.001), but data for householders were too few for testing and care should be taken in interpreting householder figures as a result. Seasonal patterns in mole control did not differ among farm enterprise types (χ^2^_(33)_ = 27.3, *p* = 0.745). Data for some categories of amenity were too few for testing.

#### 3.2.6. Mole Habitats

**Figure 5:**
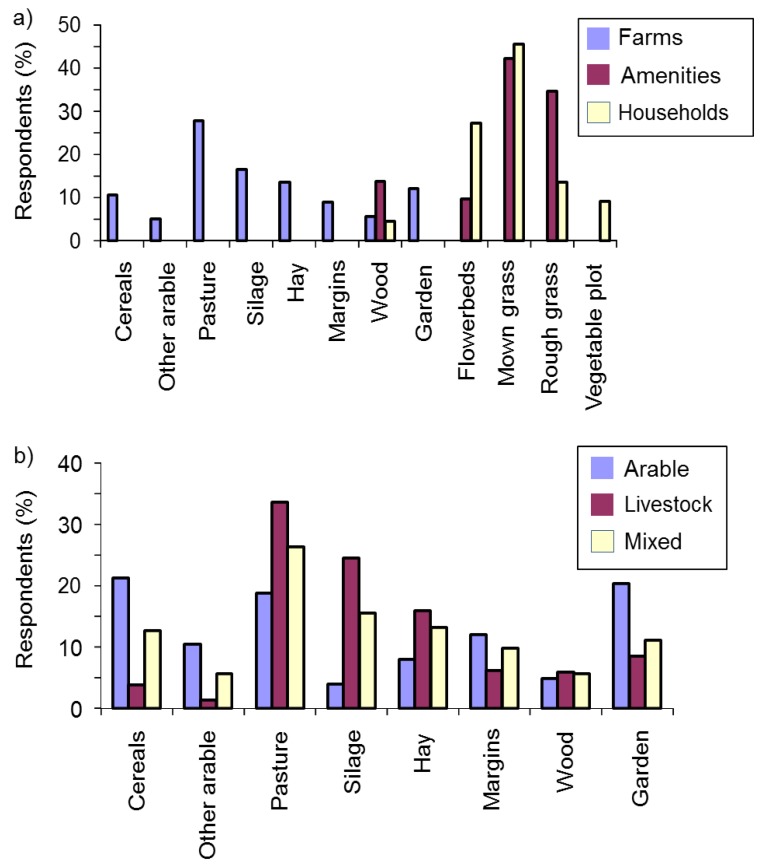
Habitats with reported mole activity in the previous year by: (**a**) respondent type (sample sizes are: farms *n* = 1735, amenities *n* = 423, households *n* = 22); (**b**) farm enterprise type (sample sizes are: arable *n* = 324, livestock *n* = 675, mixed *n* = 660).

There was no evidence for a relationship between the habitats present on respondents’ land and either mole pest status or recent mole activity, for farms (χ^2^_(8)_ ≤ 12.2, *p* ≥ 0.144), amenities (χ^2^_(4)_ ≤ 2.2, *p* ≥ 0.695) or households (χ^2^_(5)_ ≤ 2.6, *p* ≥ 0.459). However, habitats with reported recent mole activity varied among respondent types (χ^2^_(22)_ = 2188.6, *p* < 0.001). On farms, moles were most commonly reported on pasture, and grass cut for either silage or hay. On amenities they were most common on mown and rough grass, while householders reported them mostly on mown grass and flowerbeds (Figure 5a). The habitats in which moles were found also varied among farm enterprise types (χ^2^_(14)_ = 225.1, *p* < 0.001). On arable farms, moles were most likely to be reported on cereals and in gardens, while on livestock farms they tended to be on pasture or grass cut for silage, and on mixed farms they were most likely to be on pasture (Figure 5b).

Mole pest status on farms differed with the habitats in which moles were found (χ^2^_(7)_ = 26.7, *p* < 0.001). On farms where they were considered pests, moles were often reported on pasture, or grass cut for silage or hay, whereas on non-pest farms they were most often found on pasture or gardens (Figure S5). There was no evidence that mole pest status was related to the habitats in which they were found for either amenities (χ^2^_(3)_ = 1.3, *p* = 0.740) or householders (χ^2^_(4)_ = 3.2, *p* = 0.521).

#### 3.2.7. Mole Damage Types

Respondents reporting that recent mole activity had caused damage were asked to say which types of damage they had suffered. There were insufficient damage data from householders but damage types differed between farmers and amenities (χ^2^_(8)_ = 135.3, *p* < 0.001) (Table 1a). Grass or pasture being covered with soil was the most commonly reported type of damage for both groups, while farmers’ second most frequent concern was silage being spoiled. The next most common complaints for both groups were damage to machinery, damage to plants, and weed invasion on molehills.

Damage categories differed among farm enterprise types (χ^2^_(12)_ = 93.5, *p* < 0.001) with arable farmers complaining mainly of damage to plants followed by grass coverage and damage to machinery, and livestock and mixed farmers most frequently reporting grass coverage, damage to silage and damage to machinery (Table 1b). Some data categories among amenity types were too restricted for statistical analysis but all groups, except other amenities, complained most about grass coverage (Table 1c). The next most frequent concerns were: golf courses and parks, damage to machinery and weed invasion; ornamental gardens, plant damage; sports fields, injuries to people; and racecourses reported “other” concerns which were generally along the lines of damage to the racing surface presenting a risk of injury to horses and riders. There was no evidence that reported pest status varied among damage categories (χ^2^_(5)_ = 3.0, *p* = 0.700).

#### 3.2.8. Silage Production

Whether or not farmers reported making their own silage in the previous year was related to farm enterprise type (χ^2^_(2)_ = 195.3, *p* < 0.001), with 75% or more of livestock and mixed farmers, and only 15% of arable farmers making silage (Figure S6). The majority of farmers producing silage wilted the grass before baling (to allow any soil on the grass to dry and fall out) but did not raise the cutters (to avoid “scalping” the surface of the ground and incorporating soil into the silage) or use a silage additive (to reduce bacterial contamination) (Figure S7). There was no significant difference among farm enterprise types in their use of these silage protection strategies (χ^2^_(2)_ ≤ 4.7, *p* ≥ 0.096). Whether or not farmers reported silage damage by moles in the previous year was related to their use of silage protection strategies (χ^2^_(3)_ = 16.4, *p* < 0.001), with farmers that reported damage being more likely to raise the cutters or use a silage additive than those reporting no damage (Figure S8).

#### 3.2.9. Control Methods

##### Use of Control Methods

We asked respondents which types of mole control they had used in the previous year. Farmers and amenity managers were asked to choose from: gassing, harrowing/rolling, kill-trapping, live-trapping, shooting, strychnine and other. Householders were offered a similar list including rolling/flattening instead of harrowing/rolling. The vast majority of mole control was reported on farms or amenities, with kill-trapping by far the preferred method (53.2%), followed by gassing (17.0%), harrowing (13.8%), strychnine (7.3%), shooting (6.3%), live-trapping (0.8%) and other methods (1.5%) (*n* = 601) (χ^2^_(6)_ = 833.7, *p* < 0.001 for the H_0_ that methods would be equally popular). There was no evidence that choice of method differed between farmers and amenities (Fisher’s Exact, *p* = 0.360). Just six householders reported controlling moles in the previous year; rolling (*n* = 3); rolling and live-trapping (*n* = 1); live-trapping and sonic deterrents (*n* = 1); TCP-soaked rags in runs (*n* = 1).

There was no evidence that choice of control method differed between regions (Fisher’s Exact, *p* = 0.999) or among farm enterprise types (χ^2^_(10)_ = 10.9, *p* = 0.365). However relative use of the different methods did differ for some amenity types (where the frequency of methods used for each amenity type was compared to the frequency for all other amenity types combined), including golf courses, parks and other amenities (Fisher’s Exact, *p* ≤ 0.030), but not for ornamental gardens, racecourses or sports fields (Fisher’s Exact, *p* ≥ 0.614, Table 2). Nevertheless kill-trapping was used in the majority of instances by all amenity types. Racecourses and golf courses were the most likely to have used strychnine, and racecourses, parks and golf courses the most inclined to have used gassing, while parks were the most likely to have used harrowing.

We examined the relationship between reported mole damage type suffered in the previous year and the respondent’s use of strychnine, gassing or kill-trapping. There was no evidence for a relationship between these for either farmers or amenities (strychnine, Fisher’s Exact, *p* = 0.963; kill-trapping χ^2^_(6,7)_ ≥ 3.6, *p* ≥ 0.729; gassing χ^2^_(6,7)_ ≥ 5.0, *p* ≥ 0.663).

**Figure 6:**
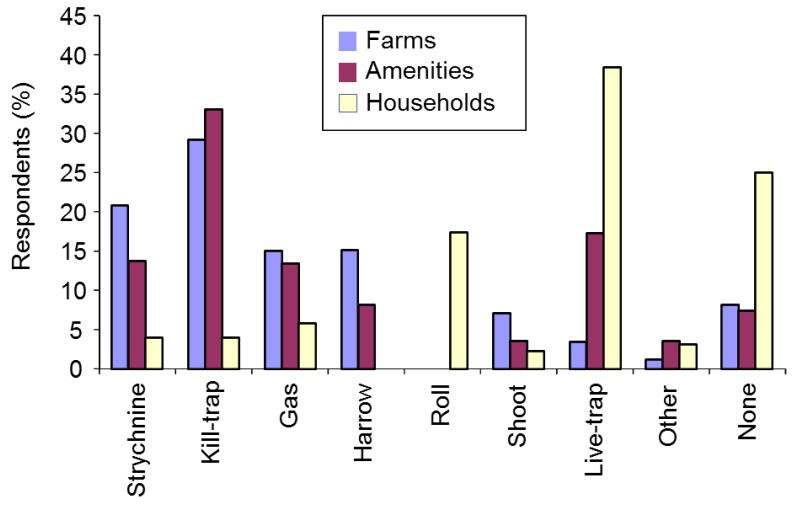
Proposed future control methods for farmers, amenity managers and householders (sample sizes are: farms *n* = 1225, amenities *n* = 487, households *n* = 224).

We asked all respondents, regardless of whether they had ever had moles on their land or controlled moles, which mole control methods they might use if moles were present on their land in future. Strychnine was included in the list of options on the proviso that it became legally available again and we also included the option of not controlling moles at all. Farmers and amenity managers differed in their choices of future control method (χ^2^_(7)_ = 133.7, *p* < 0.001). Kill-trapping remained the preferred future option for both groups, followed for farmers closely by strychnine, and then by harrowing/rolling and gassing, and for amenity managers by live-trapping, strychnine and gassing (Figure 6). Householders favoured live-trapping, no control and rolling/flattening (which they were offered instead or harrowing/rolling as before). A minority of farmers (8%) and amenity managers (7%) and 35% of householders said they would not control moles at all if they were present on their land in future.

Preferred future control methods differed among farm enterprise types (χ^2^_(14)_ = 33.0, *p* = 0.003). All farm enterprise types preferred kill-trapping, followed for livestock and mixed farmers by strychnine and for arable farmers by strychnine and gassing (Figure S9). A minority of livestock (6%) and mixed (6%) and 14% of arable farmers said they would not control moles on their land. Stated future control methods for farmers and amenity managers differed significantly between those that did and those that did not already consider moles pests on their land (χ^2^_(7)_ = 255.7, *p* < 0.001). Those thinking moles pests were most likely to choose kill-trapping, followed by strychnine and then gassing, while those who did not tended to opt for no control. Proposed future strychnine use was much more frequent where moles were considered pests than where they were not (Figure S10).

##### Strychnine Use

We examined factors affecting strychnine use in the past five years and likely future use. Where “past strychnine use” and “future strychnine use” are used here we refer, respectively, to strychnine use in the last five years and intended future strychnine use among past users if the poison became legally available again.

**Figure 7:**
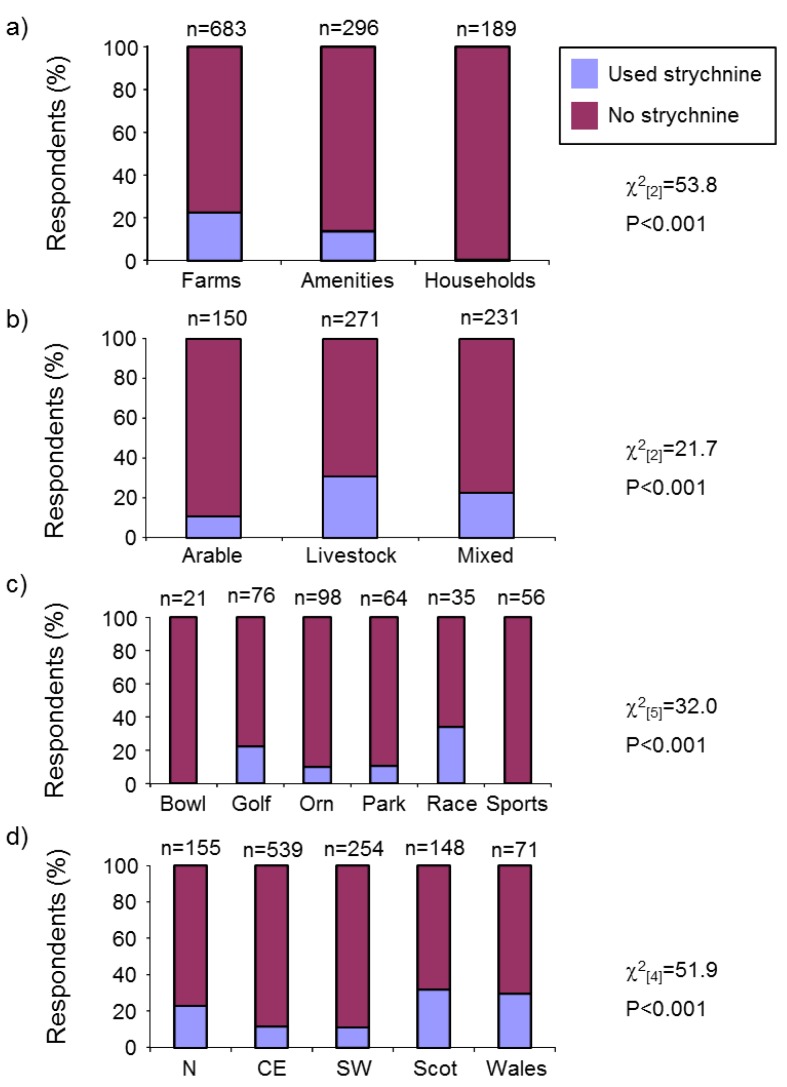
Past strychnine use by: (**a**) respondent type; (**b**) farm enterprise type; (**c**) amenity type; (**d**) region. Amenity types are: Bowl = bowling greens, Golf = golf courses, Orn = ornamental gardens, Park = parks, Race = racecourses, Sports = sports fields and Oth = other amenities. Regions are: N = northern England, CE = central and eastern England, SW = south-western England, Scot = Scotland and Wales=Wales. Statistics shown are results of χ^2^ tests.

Past strychnine use was affected by respondent type, farm enterprise, amenity type and region (Figure 7a–d), but not soil type (χ^2^_(4)_ = 4.2, *p*= 0.379). Strychnine had been used by a small proportion of farmers and amenities, and just one of 189 householders. The poison had been more commonly used in Scotland, Wales and northern England than in other parts of the country. Strychnine had been more commonly used on livestock and mixed farms than arable farms and, among amenities, was more popular on racecourses and golf courses, followed by parks and ornamental gardens. No bowling greens or sports fields reported past strychnine use.

The majority of respondents that had used strychnine in the previous five years said that they would use it on their land in future if it became legally available again. There was no evidence that this differed among farm enterprises, amenity types or regions (Figure S11b–d), but farmers were more likely (95%) than amenity managers (85%) to say that they would use strychnine again in the future. On farms and amenities that had used strychnine in the past five years, the poison was usually administered by a professional pest controller (74%) and the rest by the respondent (or, on farms, their family) or their staff, and on two farms by someone else (*n* = 204). The person that administered the poison did not differ significantly between farms and amenities (Fisher’s Exact, *p* = 1.00).

Farmers and amenity managers that had used strychnine in the previous five years differed in their choice of alternative mole control method (or decision not to control moles) after strychnine was banned (χ^2^_(4)_ = 21.1, *p* < 0.001). The main alternatives used on farms (*n* = 151) and amenities (*n* = 43) respectively were: kill-trapping (29.1% and 62.8%), gassing (11.9% and 9.3%), harrowing (10.6% and 2.3%), mixed methods (20.5% and 20.9%) and no control (27.8% and 4.7%). Kill-trapping was the option favoured by both groups but amenity managers were more likely than farmers to kill-trap and farmers more likely than amenity managers not to control moles. Favoured alternative control method was not affected by region (χ^2^_(16)_ = 18.1, *p* = 0.318), soil type (χ^2^_(8)_ = 9.5, *p* = 0.301) or farm enterprise (Fisher’s Exact, *p* = 0.968).

The majority of respondents (85%) that reported using an alternative control method, or no mole control, since the strychnine ban thought the alternative less effective than strychnine, while the remainder considered their chosen method (or no control) to be as effective, or more effective, than strychnine. Respondents’ opinions of the relative efficacy of their method compared to strychnine did not differ among methods (Fisher’s Exact, *p* = 0.31): kill-trapping (more effective 4.7%, as effective 15.6%, less effective 79.7%, *n* = 64); gassing (more 0.0%, as 13.6%, less 86.4%, *n* = 22); harrowing (more 0.0%, as 0.0%, less 100.0%, *n* = 15); no control (more 6.7%, as 0.0%, less 93.3%, *n* = 15).

##### Important Features of a Control Method

We examined which features of a mole control method respondents considered to be important. We asked all respondents to rank, from a list of features, the three that they considered most important. We then allocated a “3” to the feature ranked highest, “2” to the feature ranked second and “1” to the feature ranked third. From these numbers we produced mean relative rankings for the seven features. The features considered were: effective, cost-effective, easy to use, humane, safe for users, safe for other people and safe for non-target species.

**Figure 8:**
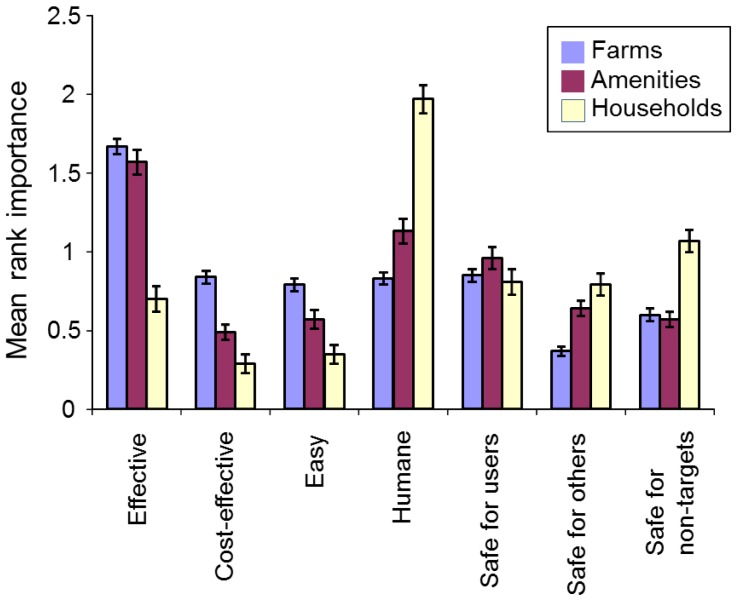
Important features of a mole control method based on mean ranked importance by respondent type (farms *n* = 629; amenities *n* = 283; households *n* = 179). Bars are means with standard errors.

Rankings differed across the seven features considered (Friedman’s χ^2^_(6)_ = 458.0, *p* < 0.001, Figure S12), with different respondent types having different priorities (Figure 8). Farmers’ three main concerns were that a control method should be effective, safe for users, and then cost-effective, whereas amenity managers felt that methods should be effective, humane, and then safe for users. Householders felt that a control method should be humane, safe for non-target species, and then safe for users.

##### Opinions about Control Methods

Respondents that had controlled moles using any method in the previous year were asked whether they considered each of a list of methods to be effective, or not, and cost-effective, or not. For farmers and amenities the list was: gassing, harrowing/rolling, kill-trapping, live-trapping, shooting, strychnine. Householders were offered a similar list including rolling/flattening instead of harrowing/rolling. However, because so few householders had controlled moles in the previous year we examined the opinions of farmers and amenity managers only.

**Figure 9:**
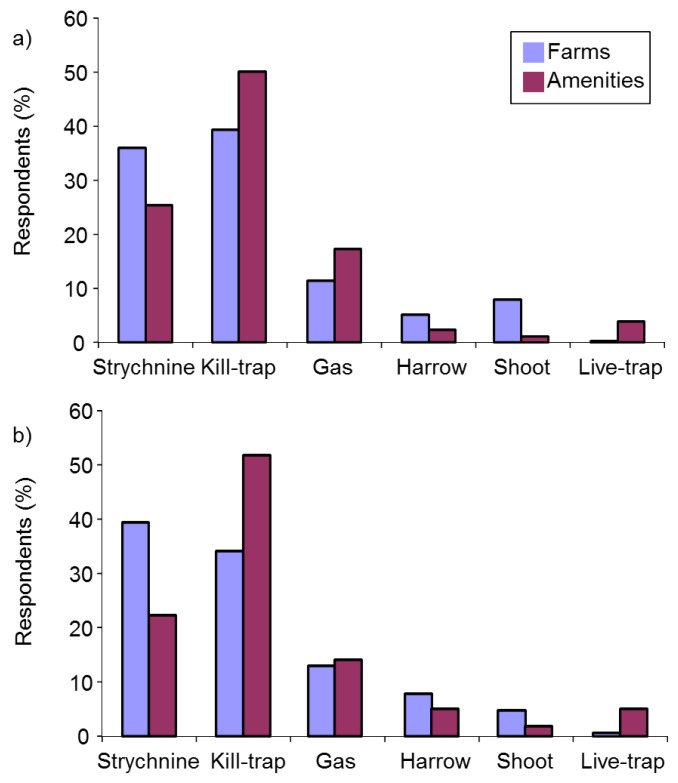
Respondent opinions of whether mole control methods were: (**a**) effective (farms *n* = 759, amenities *n* = 260); (**b**) cost-effective (farms *n* = 593, amenities *n* = 220).

Farmers and amenity managers differed in their opinions of whether certain control methods were effective and whether they were cost-effective (χ^2^_(5)_ ≥ 48.1, *p* < 0.001). Farmers were most likely to say that kill-trapping was effective, followed by strychnine and then gassing, and to say strychnine was cost-effective, followed by kill-trapping and gassing. Amenity managers were most likely to say that kill-trapping was effective, or cost-effective, followed by strychnine and then gassing. Farmers were more likely than amenity managers to say that strychnine, shooting and harrowing were effective, or cost-effective, while amenity managers were more likely than farmers to say that kill-trapping, gassing and live-trapping were effective or cost-effective (Figure 9a,b).

Farmers’ opinions of whether certain control methods were effective or cost-effective were not affected by farm enterprise type (χ^2^_(12)_ ≤ 7.3, *p* ≥ 0.840). Farmers’ and amenity managers’ opinions of whether control methods were effective or cost-effective were not related to whether they had reported recent mole damage (χ^2^_(5)_ ≤ 8.5, *p* ≥ 0.129). And while their opinions of moles as pests were not related to their opinions of whether control methods were effective (χ^2^_(5)_ = 7.3, *p* = 0.200), they were related to their opinions of whether methods were cost-effective (χ^2^_(5)_ = 18.5, *p* = 0.002). Conspicuous differences were that respondents who considered moles to be pests were more likely to say that strychnine was cost-effective and less likely to say that harrowing was cost-effective (Figure S13).

**Figure 10:**
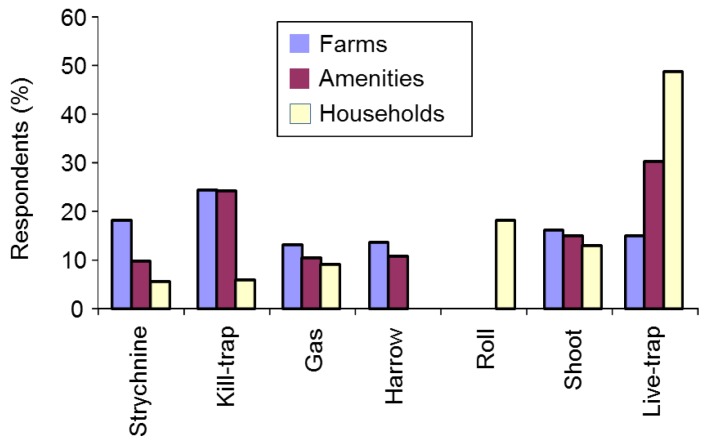
Respondent opinions of whether control methods were humane by respondent type (farms *n* = 1539, amenities *n* = 533, households *n* = 289).

We asked all respondents (regardless of whether they had moles on their land or had controlled moles before) whether they considered each of a list of methods to be humane, or not. We examined farmers and amenities separately from householders because the lists of methods offered differed between these two groups. Opinions about which methods were humane differed between farmers and amenities (χ^2^_(5)_ = 72.0, *p* < 0.001), and these also differed conspicuously to the opinions of householders where there was overlap in the methods considered (Figure 10). Farmers were most likely to say that kill-trapping was humane, followed by strychnine and then gassing or harrowing. Amenity managers were most likely to say that live-trapping was humane, followed by kill-trapping and then shooting, whereas householders were far more likely to say that live-trapping was humane than any other method.

Farmers were the group most likely to say that lethal methods were humane, while householders were the group most likely to say that live-trapping was humane. Farmers’ opinions of whether certain control methods were humane were not significantly affected by farm enterprise type (χ^2^_(15)_ = 20.0, *p* = 0.173). However, for farmers and amenities combined, opinions of whether methods were humane were related to pest status and recent mole damage (χ^2^_(5)_ ≥ 31.0, *p* < 0.001). The impact of mole pest status and damage were similar, increasing the likelihood of respondents saying that strychnine, gassing or kill-trapping were humane and reducing the likelihood of them saying the same about shooting, harrowing or live-trapping (see Figures S14 and S15).

### 3.3. Ground-Truthing

#### 3.3.1. Validation of Questionnaire Survey

First we checked whether ground-truthing participants had predicted correctly the presence or absence of molehills on their land. 82% of 33 respondents predicting mole presence, and 63% of 19 respondents predicting mole absence, predicted correctly (75% correct overall, χ^2^_(1)_ = 10.8, *p* = 0.001). One participant attributed anthills to mole damage, predicting high mole activity where there was none; he commented that he thought mole activity somehow facilitated the creation of anthills.

Next we examined whether ground-truthing participants had successfully predicted relative molehill activity, between their high and low/no molehill sites, as an indication of their ability to estimate correctly the level of mole activity measured during the 2007 questionnaire study. If our estimated molehill density (based on ground-truthing) at the site, judged (by the respondent) to have “high activity”, exceeded our estimated density for the site, judged to have low/no activity, that participant was considered to have judged correctly, and *vice-versa*. The majority of participants, 83% (*n* = 23), had judged relative molehill activity correctly between the two sites. In two of the four cases (9%) where they had not, managers had predicted relative activity levels the wrong way round, and in the other two cases (9%) there was no molehill activity at either site. Ability to judge relative activity levels correctly did not differ between farmers and amenity managers (Fisher’s Exact, *p* = 1.000) or among reported soil types (Fisher’s Exact, *p* = 1.000). Overall, sites identified by participants as having high molehill activity levels had significantly greater molehill densities (median 223 molehills·ha^−1^, mean 467 molehills·ha^−1^ (Standard Error = 188), *n* = 23) than those identified as low/no activity sites (median 0 molehills·ha^−1^, mean 55 molehills·ha^−1^ (Standard Error = 28), *n* = 29) (KW χ^2^ = 21.3, *p* < 0.001).

#### 3.3.2. Changes in Mole Activity

We took the opportunity to ask ground-truthing participants whether they felt that mole activity on their land had changed over the previous five years. Overall, more than 60% felt that it had remained the same while the remainder, that thought it had increased or decreased, were almost evenly balanced (Figure S16).

## 4. Discussion

Moles are widely controlled in Britain, but mole presence, activity and control have not been studied in detail on farmland, and have never been examined on amenities or domestic gardens. The decline (since the 1990s) in permits issued to use strychnine for poisoning moles, and its ultimate withdrawal (in 2006), were predicted to result in a dramatic increase in mole numbers, because it was claimed there was no suitable alternative control method [4,15].

Our national survey of farmers, amenity managers and householders revealed wide variation in the perceptions, opinions and behaviour of land managers regarding moles and mole control, and found no evidence of an increase in moles on farms compared to a 1992 survey [5]. There may be wider ranging implications for other policy changes regarding wildlife management. More mole control may be conducted than is needed, and much of this is done in March/April, which may be the least fruitful time of year for population control. This study provides a comprehensive and detailed characterisation of human interaction with a pest species, and a valuable benchmark as such.

### 4.1. Survey Validity

Critical to the validity of any self-selecting survey is obtaining a good response rate from an appropriate sample population [22]. Rates for postal wildlife surveys vary (e.g., 27% and 30% [24], 46% [25], 55% [9]). We designed our survey using Dillman’s Tailored Design Method [22] and, in doing so, achieved an overall response rate of 59% (farmers 63%, amenity managers 58%, householders 50%), thus securing a reasonably representative sample of the study population. Of course, we cannot rule out the possibility that self-selection biases occurred. However, ground-truthing indicated that questionnaire respondents had a good awareness of the presence or absence of moles on their land and that they were able to judge mole activity levels well.

### 4.2. Moles and Mole Control

Mole presence and mole problems were associated more with lighter than heavier soils, making sense in terms of soil workability [26], and moles have been noted to avoid stony soils [1]. Historic mole presence and control were most common on loamy and peaty soils and least common on clay, and moles were more often controlled recently (and so perhaps more regularly) on peaty and sandy soils. Mole presence was unaffected by region, but where moles were present they were conspicuously more of a concern (more likely to be considered pests, to cause damage, to be controlled) in Scotland, Wales and northern England than elsewhere in Britain. In 1992, Atkinson *et* *al*. [5] identified a similar regional effect on mole pest status, with fewer farmers in central and eastern England considering the mole a pest. They attributed this to soil “quality”, which is a rather subjective and potentially circular measure when considering mole presence. Instead we found that soil type helped to explain regional differences in mole pest status, with loamy, peaty and sandy soils being more prevalent where moles were of most concern (Scotland, Wales and northern England), and chalky and clay soils more prevalent where they were of least concern (central and eastern, and south-western England).

The vast majority of farmers and amenity managers (93% and 74% respectively), reported mole presence on their land, compared with only 13% of householders. This is probably due largely to the size of holding, with larger holdings being likely to support more moles. In 1992, in a survey of moles on British farmland, moles were reported as farmers’ third worst pest, and almost three-quarters of farmers wanted fewer moles on their land [5]. Interestingly, in a study of farmers in Wiltshire, in which farmers were asked mainly about red foxes (*Vulpes vulpes*) but asked to give the pest status of a large number of other species, only 14% reported moles as pests [27]. In the current study, most respondents with moles considered them pests (this was most pronounced for amenity managers, followed by farmers and then householders). This indicates that where moles were present, they were usually a nuisance for the land manager. There was good agreement between our 2007 study and Atkinson *et al*.’s 1992 survey [5] in that, in both, more than 90% of British farmers reported moles on their farm, and about two-thirds of farmers with moles considered them pests. We found that, among respondents with moles, farmers and amenity managers were more likely than householders to report recent activity, indicating perhaps that these groups experience more regular activity; again this is probably related to holding size. While most respondents with recent mole activity felt that this activity had caused damage, this concern was most pronounced for amenities.

Patterns of historic control were generally similar to those for historic mole presence, but occurring at a lower level, indicating that 28% of farmers, 15% of amenity managers and 7% of householders had moles at some point but opted not to control them. Nevertheless, most farmers and amenity managers had controlled moles at some time and the majority of these had controlled moles recently (and so perhaps regularly); again, this was most conspicuous for amenities.

Farmers with livestock or mixed holdings were more likely than arable farmers to report mole presence, problems and control. This was probably related to enterprise type and, for example, related damage to pasture or silage, rather than size of holding, as livestock and mixed farms tend to be smaller than arable. Soil type may also have been a factor as, for example, more arable farming occurs in eastern and southern England (where heavier soils are more prevalent), than in other parts of Britain. Among amenities, it is not surprising that smaller types, e.g., bowling greens and sports fields, were less likely to report mole presence. Of amenities with moles, managers of large, high value enterprises (golf courses and racecourses), almost unanimously considered moles pests and were more likely to have controlled them.

In 1992, farmers who considered moles pests reported a mean loss due to mole activity and control of £126 (or £235 at 2015 levels [8]); no distinction was made between the costs of damage and control [5]. In 2007, we gathered separate costs data for damage and control from those respondents reporting recent damage or control, respectively. When our mean costs were combined, these equated to £866 for farmers, £1050 for amenities and £187 for householders (£1084, £1314 and £234 at 2015 levels respectively). If we compare 2015-level costs from farms in 1992 (£235) and in 2007 (£1084), the figures are vastly different. This could suggest that mole problems have worsened. However, our questionnaire data indicate that there was no change in the proportion of farmers affected by moles between 1992 and 2007, and our ground-truthing participants felt overall that mole activity on their land had stayed the same between 2002 and 2007. There are three other possible and potentially overlapping explanations for the several-fold difference between the 1992 and 2007 cost figures: (1) while it seems that mole activity has not spread, it may have intensified in some places; (2) while mole control efforts may have stayed the same, control may have become more expensive with the demise of the relatively inexpensive option of strychnine; (3) the differences may be largely due to the way data were collected. Atkinson *et al*. gathered losses data only from farmers who considered moles pests. However, in their study, 31% of farmers who were indifferent to moles killed moles anyway and their losses will not have been taken into account. Because we collected costs data from all respondents reporting damage or control, and because we sought damage and control costs separately, and included time spent on non-professional control, our respondents are likely to have given a more fully considered answer, perhaps reflecting more accurately the true cost of mole damage and control.

Control costs per holding were greatest for amenities, farms and then households. This is likely to reflect the larger size of farms and amenities compared to domestic gardens, so we calculated costs per hectare and found costs of both damage and control to be greater for gardens, amenities and then farms. This may reflect the intensity of attention and cultivation per unit area invested in domestic gardens and some amenities. Among farms and amenities, the greatest costs of damage and control per hectare were attributed to livestock farms and race-courses respectively; this reflects our findings that these were the farm and enterprise types reporting most mole problems, and Atkinson *et al*.’s result that financial losses were greatest for farms consisting primarily of pasture [5]. Other amenities where relatively large amounts were spent on mole control were ornamental gardens and three sports fields.

In 1981, Mellanby proposed that damage by moles did not justify economically the effort spent controlling them [28]. For each of farms, amenities and households, we found that a larger number of respondents reported control costs than reported damage costs, indicating some may have spent money prophylactically or to rectify damage that could not be attributed a financial value. Amenity managers spent more money on control than the value of damage they sustained, while farmers and the few householders answering the question sustained a greater value of damage than they spent on control, suggesting that some amenities may be less prepared than other respondents to tolerate mole activity; this may be because conspicuous mole presence is felt to have a direct impact on the commercial success of such amenities. Of course, it is impossible to say how much mole damage is being suppressed by control activities or whether more control is conducted than is necessary. However, if managers cannot value the mole damage they sustain or what they save by acting proactively, then controlling moles on their land may not be economically, or potentially ethically, justified.

Moles are found on a wide range of habitats, including grassland, permanent pasture, arable land, gardens, woodland (both deciduous and coniferous), fenland, breck and the uplands [3,26]. Mole damage reported here spanned a wide variety of issues relating to the particular habitats and activities occurring on the land in question. For example, farmers mostly reported moles on pasture and on grass cut for either silage or hay, amenity managers on mown and rough grass, and householders on mown grass and flowerbeds. Coverage of grass or pasture with molehill soil was the most commonly reported damage for both farms and amenities, and reports have been documented of extreme cases where 4, 7 and 11% of pasture land was covered [29], but the frequency of such occurrences is not known.

In 1994, Atkinson *et al*. proposed that the most important single mole-related problem on farmland might be the pollution of silage (a fodder of fermented grass used to feed livestock when pasture is in short supply [5]), but this was farmers’ second greatest concern in our study. Silage may become polluted if soil bacteria, such as *Clostridium* spp., are gathered up with grass when it is cut and bagged for silage, and polluted silage may be less nutritious and inedible [5]. Silage can also be contaminated with *Listeria monocytogenes*, from the soil, and this may cause listeric encephalitis in sheep, cattle, goats and pigs [30]. Silage protection measures are available; we found that most silage producers allowed cut grass to wilt before bagging (encouraging soil to dry and fall out, a no-cost option), but only a minority raised the grass cutters to avoid “scalping” molehills and other raised areas (raising cutters can reduce yield by approximately 5%), or applied a silage additive designed to control pollution (Atkinson *et al*. reported that this reduced neither the amount lost, nor that rendered sub-standard [5]). We found that farmers reporting mole damage were more likely to have raised the cutters or used a silage additive (the options costing money) than those reporting no damage. It is not possible to say whether these silage protection measures failed, or whether farmers using these measures sustained less silage damage than they would have done without them.

Seasonal patterns in reported mole activity and control may, at least partly, reflect the interests and activities of respondents, rather than the pattern of mole activity, with farmers and amenity managers reporting a large activity peak in spring and a smaller one in autumn, and householders reporting a single peak in summer. Also mole signs are more conspicuous on some habitats than others [5]. Atkinson *et al*. [5] found a similar pattern for farms in 1992, but our autumn peak was considerably smaller. There is likely to be a genuine peak in mole activity in spring when male moles tunnel more in search of mates [2] and a surge in summer when young moles disperse from natal nests, the precise timing depending on food availability in the maternal territory [31]. However, the number of molehills is not a good indicator of the number of moles, but simply of the amount of digging occurring. When soil is drier in the summer, moles forage in deeper tunnels and excavate less because the ground is harder and more difficult to dig, and tunnels are less likely to collapse. They dig more if tunnels become damaged through freezing and thawing, or unusable due to flooding [1], potentially explaining observed autumnal activity.

### 4.3. Mole Management Methods

Before strychnine was withdrawn it was considered the most cost-effective method of large-scale mole control in Britain and it was suggested that, in its absence, managers would turn to kill-trapping [4,5]. Indeed, we found that strychnine was the method most often considered cost-effective among farmers, while kill-trapping was by far the most popular method of mole control on farms and amenities in the year strychnine withdrawal occurred (the year before the 2007 survey). Kill-trapping was also farmers’ and amenity managers’ favoured future control method, but strychnine was a popular second favourite among farmers, with 21% saying they would use it again if it became available in future (and 29% saying they would use kill-trapping), and third choice among amenity managers. Our findings regarding kill-trapping were corroborated by the fact that farmers and amenity managers agreed that effectiveness was the most important feature of a mole control method, and kill-trapping was the method most often thought effective by both groups. However most mole control in this study was reported in March and April, which may be the least effective time to trap moles, because moles caught during this period tend to be breeding males [32,33]. Farmers and amenity managers may therefore be expending unnecessary effort killing male moles in the spring. Mole trapping for long-term population control might be better targeted outside the peak in male breeding activity, when females are more likely to be caught, but this could threaten the welfare of young moles if conducted when they are still dependent [32].

It is intriguing that while amenity managers’ second most often used method in the previous year was gassing and their seventh most often used method was live-trapping, their second most preferred future method was neither gassing nor strychnine, but live-trapping. Reasons for this difference are not clear, but it may reflect a disparity in what amenity managers do and what they would like to do. For example, perhaps they would have liked to use live-trapping (the method they most often considered humane), whereas effectiveness had to be the priority (see above), because amenity managers are generally employees rather than owners. Also, several amenity managers mentioned that they were restricted in terms of the methods available to them because some were impractical or potentially dangerous because of public access. In some cases the organisation had a policy of not using certain methods, e.g., strychnine, gassing, so this is likely to have influenced both their previous and proposed control methods.

Only six households controlled moles in the previous year, mainly using rolling or live-trapping; one used TCP**^®^** as a repellent, which is not registered for this purpose and therefore not a legal means of controlling moles. Householders considered humaneness the most important aspect of a control method; the method they most often thought humane was live-trapping, and live-trapping was overwhelmingly the most popular future control method chosen by this group. Respondents that considered moles pests were most likely to choose lethal future methods (kill-trapping, strychnine and then gassing), while respondents who felt moles were not pests said that (in the hypothetical scenario where they had moles on their land) they were most likely to opt for no control, harrowing and then kill-trapping.

Respondents’ opinions about whether various control methods were effective, cost-effective or humane varied widely. Opinions were most likely influenced by respondents’ own experiences of mole management methods and moles, and their attitudes towards the species in general. Differences in opinions about humaneness probably also reflect the facts that there is no accepted definition of “humane”(see [34]) and that mole poisoning and trapping occur underground where people cannot see how they die. The delayed death and muscle spasms associated with strychnine poisoning are acknowledged to indicate non-humane control.

Unfortunately live-trapping may not offer the humane mole management option that many respondents probably hope [35]. Moles have a high metabolic rate and are poorly disposed to live-trapping (potentially dying from cold, wet, starvation and “battering themselves” against the walls of old-fashioned wooden Friesian traps [26]), so it is recommended that traps are provisioned with food [36] and checked every 4–8 h [2,26]. However, live traps are unregulated and conditions inside the only live mole trap currently available “off the shelf” in Britain, the plastic “mole tube trap”, are far from ideal (narrow, cold and likely to be damp, without room for bedding or insulating air, allowing multiple captures likely to lead to fighting) and not recommended [35]. Most users of live mole traps probably release captured moles but Natural England does not recommend this on welfare grounds; release into an existing territory is likely to lead to fighting with the resident mole, while release into an area without an existing tunnel system could commit the mole to starvation before it is able to build a tunnel system of its own and could constitute an offence under the Animal Welfare Act 2006 [36].

Kill-trapping, the most popular form of mole control, was the method most often thought humane by farmers, and second most often (after live-trapping) by amenity managers and householders. Some kill-traps may kill moles quickly and relatively humanely. However, this has not been tested because mole kill-traps are exempt from welfare approval requirements under the 1954 Pests Act, since The Small Ground Vermin Traps Order 1958 permits their use. Probably because of this exemption, Baker *et al*. (2012) found that the mechanical performance of mole kill-traps varies widely, and they suggested that the welfare performance of at least some of these traps may be in doubt [32,37]. The welfare impact of mole traps should be tested and evaluated according to the trap standards to which other spring traps are subjected in the UK [37].

A minority of respondents had used strychnine in the previous five years (farmers 22%, amenity managers 14% and householders 0.5%). Strychnine was used more often by respondents identified as having more mole problems in general, e.g., farmers and amenities, respondents from Scotland, Wales and northern England, livestock and mixed farms, racecourses and golf-courses. Most previous strychnine users said they would use it again if it became available in future. Among previous users, farmers tended to be keener than amenity managers to use strychnine again (potentially because it was the method farmers, but not amenity managers, most often thought cost-effective). Kill-trapping was the strychnine alternative most frequently used by previous strychnine users; others used gassing, harrowing or no control. 85% of respondents using an alternative to strychnine thought the alternative less effective. Before strychnine was withdrawn it was predicted that its withdrawal would cause a dramatic increase in mole populations [4,18], and afterwards it was claimed that it had done so [19,20,21]. However, we found no evidence for a spread in mole presence or an increase in their pestilence between 1992 and 2007.

## 5. Conclusions

Respondents perceiving the greatest mole problem were amenity managers (especially on racecourses and golf-courses), farmers (especially of livestock and mixed enterprises) and respondents in Wales, Scotland and northern England. Coverage of grass or pasture, and damage to silage, were the most frequently reported concerns. Kill-trapping was overwhelmingly the most frequently used control method in the year following strychnine withdrawal and was also the most often proposed future control method, even if strychnine were to become available again. However, strychnine was a popular second and third choice for future control among farmers and amenity managers, respectively, and most previous strychnine users said they would use it again in future if it became available. More research is needed on the actual losses incurred and on the effectiveness of mole control methods and silage protection measures in resolving these. Amenities spent more money on mole control than the value of any damage sustained and may be the group for whom moles are the greatest concern. In general the financial costs of mole damage and control may be considerably greater than have been reported previously, but nevertheless small by comparison to those, for example, associated with badger damage caused to crops, or through burrowing or predation.

Despite predictions and claims relating to dramatic rises in mole populations following strychnine withdrawal, more farmers and amenity managers said they would use kill-trapping over strychnine in future if strychnine became available again. They reported that effectiveness was the most important factor in choosing a mole control method and that kill-trapping was the most effective method. Humaneness was also important to amenity managers and to householders, and live-trapping was their second-favourite and favourite future method, respectively. However, live-trapping may not offer the humane solution they hope for. More information is needed on the humaneness of control methods. More mole control may be carried out in Britain than is necessary, and much of this seems to happen in March/April, the least fruitful time of year for population control. Before controlling moles, land managers should think carefully about whether this can be justified and about which method, if any, they use (see [38]). An Integrated Pest Management programme, prioritising proactive rather than reactive measures (e.g., education, collaboration, activity monitoring and properly targeted management, as has been recommended for Northern pocket gophers and Richardson’s ground squirrels) would be beneficial [16]. In many cases it may be sufficient to harrow and roll molehills, when necessary, to distribute soil and protect silage crops, pasture and other grassland. On high value amenities, where molehills and tunnels cannot be tolerated, targeted trapping might be the best option, with moles being removed as and when signs occur. Avoiding the breeding period would maximise effectiveness and minimise welfare impacts. Moles have been treated unequally to most other species in wildlife management, with a lower welfare threshold tolerated as evidenced by the use of strychnine and unregulated traps [37]. It seems that the withdrawal of strychnine has not caused the large rise in mole numbers that was forecast, and there may be wider ranging implications, e.g., the regulation of currently unregulated mole (and rodent break-back) traps might be considered acceptable.

## Figures and Tables

**Table 1:** Percentage of respondents reporting mole damage of each type by: (a) respondent type (farms and amenities only); (b) farm enterprise type; (c) amenity type.

Response Group	Grass	Silage	Machinery	Plants	Weeds	Water Courses	People Injured	Animals Injured	Other Damage	*n*
(a) Respondent types											
Farmers	29.7	24.1	16.2	10.0	12.6	3.2	0.0	0.6	3.7	848
Amenities	45.4	0.0	15.4	11.1	12.4	4.0	3.1	0.0	8.6	324
(b) Farm enterprise types											
Arable	22.6	3.8	19.8	30.2	13.2	2.8	0.0	0.0	7.6	106
Livestock	32.0	29.3	13.7	5.0	12.7	4.2	0.0	0.0	3.2	403
Mixed	30.4	25.7	18.2	9.8	12.5	2.0	0.0	0.0	1.4	296
(c) Amenity types											
Golf courses	43.6	0.0	21.4	6.0	16.2	6.8	1.7	0.0	4.3	117
Ornamental gardens	49.5	0.0	10.8	19.4	8.6	1.1	5.4	0.0	5.4	93
Parks	43.5	0.0	15.2	10.9	21.7	0.0	0.0	0.0	8.7	46
Racecourses	44.2	0.0	14.0	9.3	4.7	4.7	0.0	0.0	23.3	43
Sports fields	61.5	0.0	7.7	0.0	0.0	7.7	15.4	0.0	7.7	13
Other amenities	14.3	0.0	14.3	14.3	14.3	0.0	14.3	0.0	28.6	7

**Table 2:** Percentage of respondents reporting that they conducted mole control using each method in the previous year by amenity type.

Amenity Type	Gas (%)	Harrow (%)	Kill-Trap (%)	Live-Trap (%)	Shoot (%)	Strychnine (%)	Other (%)	*n*
Golf courses	18.0	0.0	72.1	1.6	1.6	6.6	0.0	61
Ornamental gardens	13.9	4.6	73.9	0.0	1.5	4.6	1.5	65
Parks	22.5	17.5	52.5	0.0	2.5	2.5	2.5	40
Racecourses	23.7	7.9	57.9	0.0	0.0	7.9	2.6	38
Sports fields	10.0	0.0	80.0	0.0	0.0	0.0	10.0	10
Other amenities	15.8	10.5	52.6	0.0	10.5	0.0	10.5	19

## Supplementary Materials

The following are available online at http://www.mdpi.com/2076-2615/6/6/39/s1, Text S1: Questionnaire survey method, Text S2: Example contact 1, pre-notice letter, Text S3: Example contact 2, letter accompanying questionnaire, Text S4: Example Questionnaire, Text S5: Example contact 3, reminder / thank you card, Text S6: Example contact 4, letter accompanying replacement questionnaire, Text S7: Ground-truthing survey method, Text S8: Ground-truthing data manipulation and analysis, Text S9: Questionnaire response rates, Text S10: Factors affecting recent mole activity and damage, Table S1: Questionnaire responses for farmers, amenity managers and householders, Figure S1: Response rates by region (CE = central and eastern England *n* = 1070, North = northern England *n* = 310, SW = south-western England *n* = 383, Scotland *n* = 259, Wales *n* = 123), Figure S2: Soil type by region (N = northern England *n* = 124, CE = central and eastern England *n* = 433, SW = south-western England *n* = 202, Scot *n* = 122, Wales *n* = 45), Figure S3: Recent mole activity and mole damage, respectively, by: (a, b) respondent type; (c, d) farm enterprise; (e, f) amenity type; (g, h) region; (i, j) soil type. Regions are: N = northern England, CE = central and eastern England, SW = south-western England, Scot = Scotland and Wales = Wales. Statistics shown are results of χ^2^ tests, Figure S4: Historic and recent mole control by: (a, b) respondent type; (c, d) farm enterprise; (e, f) amenity type; (g, h) region; (i, j) soil type. Regions are: N = northern England, CE = central and eastern England, SW = south-western England, Scot = Scotland and Wales = Wales. Statistics shown are results of χ^2^ tests, Figure S5: Habitats with reported mole activity in the previous year by farm mole pest status (sample sizes are: pest *n* = 1345, not pest *n* = 338), Figure S6: Silage production by farm enterprise type (arable *n* = 150, livestock *n* = 272, mixed *n* = 228), Figure S7: Silage damage mitigation measures taken by enterprise type: (a) wilting grass before baling (arable *n* = 22, livestock *n* = 213, mixed *n* = 171); (b) raising cutters (arable *n* = 23, livestock *n* = 213, mixed *n* = 171); and (c) using a silage additive (arable *n* = 22, livestock n = 213, mixed *n* = 171), Figure S8: Reported silage damage by moles and silage protection measures taken by farmers (silage damage *n* = 253, no silage damage *n* = 212), Figure S9: Proposed future control methods by farm enterprise type (sample sizes are: arable *n* = 231, livestock *n* = 476, mixed *n* = 426), Figure S10: Proposed future control methods on farms and amenities by mole pest status (sample sizes are: pest *n* = 1181, not pest *n* = 290), Figure S11: Intended future strychnine use by: (a) respondent type; (b) region; (c) farm enterprise; (d) amenity type. Regions are: N = northern England, CE = central and eastern England, SW = south-western England, Scot = Scotland and Wales = Wales. Statistics shown are results of χ^2^ tests, Figure S12: Important features of a mole control method based on mean ranked importance for all respondents (*n* = 1091), Figure S13: Respondent opinions of whether mole control methods were cost-effective by mole pest status (farmers and amenities only; pest *n* = 716, not pest *n* = 72), Figure S14: Respondent opinions of whether control methods were humane by mole pest status (farmers and amenities only; pest *n* = 1416, not pest *n* = 396), Figure S15: Respondent opinions of whether control methods were humane by mole damage status (farmers and amenities only; damage *n* = 1203; no damage *n* = 501), Figure S16: Ground-truthing participant opinions regarding recent changes in mole activity on their land over the previous five years (farms *n* = 17, amenities *n* = 8, households *n* = 4).
