# Supplementary material for: Moles and Mole Control on British Farms, Amenities and Gardens after Strychnine Withdrawal
Source: Animals (Basel). 2016 Jun 8;6(6):39. doi: 10.3390/ani6060039 (PMC4929419; doi:10.3390/ani6060039)
Supplement: Supplementary file 1 [file animals-06-00039-s001.zip › Text S7.docx]

**Text S7: Ground-Truthing Survey Method**

We devised a two-stage process consisting of a brief face-to-face interview with participants, followed by a field survey of sites identified and discussed during the interview. Ethics approval was granted by the Central Universities Research Ethics Committee at the University of Oxford on the grounds that we obtained written consent from participants to use their interview data by obtaining their signature at the foot of the questionnaire.

*Selecting and Contacting Participants*

We selected potential ground-truthing participants from questionnaire respondents who had indicated an interest in taking part in a follow-up study. We used our Access database to identify a representative sample of questionnaire respondents as potential candidates for ground-truthing. We targeted key respondent groups (farmers, amenity managers and householders) to reflect the proportions in which they responded to the questionnaire. Ideally ground-truthees would have been stratified geographically across Britain but ground-truthing had to be restricted to the Thames Valley area because of time and financial constraints. We wrote to potential candidates a few weeks before we hoped to interview them, forewarning them that we would be phoning them to ask if they were prepared to take part. A couple of days after sending the letters we began intensive phoning of potential participants to discuss the work, and where possible to arrange a face-to-face interview. When we arranged interviews with farmers or amenity managers we checked with them the extent of their land so that we could prepare maps and aerial photos to take to the interview.

In total we ground-truthed 29 respondents representing farmers (*n* = 17), amenity managers (*n* = 8) and householders (*n* = 4) in the proportions 59%, 28% and 14%, which closely reflected the proportions in which they responded to the original questionnaire (57%, 24%, and 19%). The amenities comprised three ornamental gardens, two golf clubs, one racecourse, one ancient monument, and one park. The farms included seven mixed, six livestock, two arable, and two other farms.

*Face-To-Face Interviews*

In advance of each interview we prepared and printed maps and aerial photos of the participant’s land at various scales. Semi-structured interviews were conducted face-to-face on the participant’s premises and took between 30 minutes and an hour.

The primary purpose of the interview was to identify sites for subsequent ground-truthing (using field survey). Questions were read out to the participant. We started by asking the respondent whether there were molehills or mole tunnels on their land at the time of the interview. If they said yes, we asked them to identify, on maps and aerial photographs, six sites (fields/areas) on their land which had at that time the greatest (3 sites) and least (3 sites) mole activity (“high activity” and “low activity” sites respectively). Where a respondent reported no current mole activity on his land, he was asked to nominate three “no activity” sites. We also gathered information associated with the nominated sites, e.g., current land-use (e.g., pasture, cereal, mown grass *etc*) and site access points.

At the end of the interview we agreed a suitable day on which to conduct field surveys and discussed access *etc*. Field surveys were conducted as soon as possible (within a few days) after the face-to-face interview to maximise the currency of the interview information.

*Field Surveys*

In most cases we selected two sites (one “high activity” and one “low activity”) on each participant’s land for field survey. In selecting sites we attempted to balance the representation of different field/area types as well as taking into account accessibility and practicality. Within this remit we also prioritised sites with the most and the least activity. Where a participant reported no mole activity we surveyed one of their nominated ‘no activity’ sites.

We designed a simple survey strategy that could be adapted to different field circumstances; this involved conducting a number of point surveys (at least 30) at each site using distance sampling techniques or circular sample plots (depending on circumstances) to record molehill activity at each full or half survey point.

*Field Equipment*

We used a survey-grade hand-held GPS with sub-metre accuracy (Magellan Professional Mobile Mapper CX), and associated software (Digiterra Explorer 5). Together these allow the user to plot areas and points and to visualise them on a map displayed on the GPS screen as the data are collected. The layer files produced can then be converted to shape files for use with ArcGIS and other GIS packages. We also used a digital camera, a sighting compass for measuring angles and a laser measurer for measuring distances.

*Survey Procedure*

First we recorded basic site data, including site name, date, original questionnaire ID, type (e.g., farm, amenity, or household), participant name, main habitat (e.g., cereal, pasture, mown grass, rough grass *etc*), participant’s site name, and reported mole activity (high/low/none). We also recorded the survey start and finish times, and ticked boxes to say that the access point location (the access point was the point nearest to the access at which it was practical to take panoramic photos) had been logged using GPS, the survey site photographed from the access point using a series of over-lapping pictures (taken left to right, to cover 360^°^), and the site perimeter mapped (using GPS to create a GIS area layer file). On a few amenities and gardens the site contained discrete patches of different habitats, e.g., flowerbeds, wooded areas, sports pitches *etc*, as well as the main habitat type, in which case we mapped the main habitat patches using a separate GPS area layer for each type.

The remainder of the survey involved recording molehill activity data gathered at points along a number of straight-line transects across the site. After plotting the site perimeter, we made an assessment (based on GPS data gathered on the size of the site) to decide on a number of transects and the spacing of survey points needed to achieve at least 30 full-point plots (2 half-points = 1 full-point) across the site. If, when plotting the site perimeter we noticed that molehills were very patchily distributed, we included extra transects/plots. In general we aimed to construct several main transects across the width of the site (unless this was impractical, e.g., where tramlines within crops travelled lengthwise, in which case we followed some of these as transects), as well as one along each of the near and far boundaries (short ends).

Each main transect began and ended with a half-point plot and a series of full-point plots was spaced approximately evenly between these along each transect (unless a distinct habitat transition occurred partway along a transect, e.g., from field margin to wheat crop, in which case we placed two half-point plots (back-to-back) at the transition point. The near and far boundary transects consisted of a series of half-point plots (omitting the site corners). Each of the main transects were walked in turn, and numbered 1, 2, 3, *etc*, followed by the two boundary plots, which were named “near boundary” (nearest main transect 1) and “far boundary” (nearest last main transect). The first transect was begun at the first half-point plot on the long boundary. We recorded the transect number or name, the start location (x/y co-ordinates) and boundary habitat. We recorded the transect angle using a sighting compass (standing with our back to the long boundary). Point surveys were conducted at all full and half-point plots using either circular sample plots or distance sampling. We decided which method to use, after exploring the site, based on the answer to the question: “*Is the main habitat such (e.g., short, homogenous grass) that I can see all likely molehills within a 10 m radius of me*?” If the answer was yes, we used circular sample plots and if the answer was no, we used distance sampling.

*(i) Circular Sample Plots (Circular Plots, CP)*

Each full-point plot used as circular sampling plots had a radius of 10 m and we ensured no overlap between adjacent points. If a habitat transition occurred so close to the boundary that there was risk of overlap between for example two half-point plots (boundary and habitat transition) points, then we either omitted the overlapping habitat transition half-point, or where possible, staggered the habitat transition points sideways so that they did not overlap with the others. Details of each plot were recorded on a new line in the main body of the recording sheet. Survey points were numbered continuously from 1 for the whole site. The position of all survey plots was fixed using GPS to create a GIS “points” layer file for the site. We recorded the survey method (in this case CP) and indicated whether or not the point lay on a habitat transition. If the plot fell in a habitat other than the main one (e.g., field margin rather than crop) then we specified the secondary habitat type. We indicated whether we were conducting a half-point or full-point survey.

At each full and half-point plot we recorded the approximate mean vegetation depth for the plot using the laser measurer. If the plot contained bushes/shrubs/trees we estimated what proportion of the plot they took up and recorded this in the column “% plot taken up by shrubs *etc*”, and recorded the mean vegetation depth for the rest of the plot only. Then we searched the full or half-point plot for molehills - standing at the survey point and rotating slowly in a circle or semicircle respectively, looking for molehills within a 10m radius. We recorded either 1 or 0 in the column “Molehill obs” to indicate whether molehills were detected. For each molehill detected we used a compass to measure the angle and the laser measurer to determine the distance from the survey point, using a separate line in the datasheet for each molehill. Where there were ≥10 molehills within the radius of a full-point plot (≥5 in a half-point), we recorded only the total number of molehills seen rather than the distance and angle of each. On reaching the end of the transect we recorded the finish boundary habitat.

We moved on to each transect in turn, starting a new sheet (and recording transect details) for each. After completing the main transects we surveyed the near and far boundary transects. For each of these, we recorded the main boundary habitat under “start boundary habitat” and nothing was recorded under “finish boundary habitat”.

*(ii) Distance Sampling (DS)*

DS techniques were used if molehill detection within 10 m declined with distance from the observer, basically all situations other than very short, homogenous vegetation. This had three main implications for the survey method, which was otherwise identical to that for circular plots:

- Rather than searching for molehills in a 10 m radius plot or half plot, this method involved searching for and recording molehills up to whatever distance they could be seen from the centre of the plot, whether this was greater or less than 10 m.
- We had to take into account the likely maximum detectable distance when planning the position and frequency of survey points (to avoid overlap). In a very patchy habitat it may be possible to see some molehills quite a distance away (>>10 m), in which case it may not be possible to achieve 30 full-point surveys while avoiding overlap.
- Where the survey area met a secondary habitat, molehills were only recorded in the primary habitat.

As with circular sampling plots, if there were ≥10 molehills (for a full-point plot, or ≥5 in a half-point) within the field of view, we recorded the total number of, and distances to, molehills but not the angles.

*(iii) Combining Methods*

At some sites it made sense to combine the two survey methods, in which case the survey point spacing needed to vary to ensure no overlap.
