# Supplementary material for: Moles and Mole Control on British Farms, Amenities and Gardens after Strychnine Withdrawal
Source: Animals (Basel). 2016 Jun 8;6(6):39. doi: 10.3390/ani6060039 (PMC4929419; doi:10.3390/ani6060039)
Supplement: Supplementary file 1 [file animals-06-00039-s001.zip › Text S8.docx]

**Text S8: Ground-Truthing Data Manipulation and Analysis**

The GPS survey layers were converted to GIS shape files, imported into ArcGIS and overlaid on OS map tiles. We analysed the field survey data using SAS statistical software. First we explored the data gathered from DS plots to determine whether there were sufficient observations to use DS methods for analysing these data. DS requires approximately 60 observations (survey points) per “detection function” class, a class representing the probability of detecting the study subject in a certain habitat, e.g., pasture with vegetation height 20–30 cm. In this case, the delineation of these classes depends on how molehill observability changes with vegetation height, and we judged that 10 cm class intervals were appropriate. Combining all DS plots across all sites, we identified 13 detection function classes, covering three habitat types (mown grass, pasture/silage, and rough grass) and various 10 cm vegetation depth intervals. Each of these detection functions was represented by between 1 and 64 observations. We considered broadening vegetation height categories (hence combining certain detection functions) in an effort to achieve sufficient observations for each detection function but decided this could not be done without compromising the reliability of the data. Instead we treated DS plots as circular plots by defining the radius of the plot as the furthest distance at which a molehill was observed (to accommodate the fact that DS plots were conducted where molehill observability declined with distance). At survey points where no molehills were recorded we used the mean maximum observation distance from plots at the same site where molehills were observed.

For each survey point we divided the total number of molehills observed by the total sampling area to obtain an estimate of molehill density. Then we calculated the mean density per site across all survey points (regardless of whether they had originally been CP or DS plots), together with the standard error, and upper and lower confidence intervals. We analysed the GT data using SAS to determine whether land managers had correctly attributed damage to moles, and whether they had successfully estimated the relative severity of damage on their land.
