# Supplementary material for: Moles and Mole Control on British Farms, Amenities and Gardens after Strychnine Withdrawal
Source: Animals (Basel). 2016 Jun 8;6(6):39. doi: 10.3390/ani6060039 (PMC4929419; doi:10.3390/ani6060039)
Supplement: Supplementary file 1 [file animals-06-00039-s001.zip › Text S9.docx]

**Text S9: Questionnaire Response Rates**

Return dates were recorded for all questionnaires returned. These ranged between 2 July and 11 November. A number of questionnaires were returned undelivered (address unknown, addressee unknown or gone away), and we were notified that some other recipients were deceased or retired from farming. In some cases questionnaires were returned by respondents who had been in their respective role (farmers, amenity manager or householder) for less than a year. All such potential respondents were excluded from the sample.

After excluding these recipients, our potential sample sizes were 1143 farmers, 526 amenity managers, and 481 householders (see Table S1). Of these, we received usable responses from 63% of farmers, 58% of amenity managers, and 50% of householders (χ^2^_[2]_=22.9 *p* < 0.001)).

A number of recipients either returned blank questionnaires (as directed if they did not wish to take part in the survey) or contacted us to say that they did not wish to participate. These accounted for a small proportion of our potential sample (farmers 5%, amenity managers 2%, and householders 3%). The remaining 32% of farmers, 40% of amenity managers, and 47% of householders in the potential sample did not respond in any way.

Our response rates (63%, 58% and 50%) are considered good for self-administered surveys, particularly for those where there is no legal obligation to participate [1]. Response rates were considerably greater than we expected, particularly for the farmer group. Handling and logging the large number of returned questionnaires and entering the data into Microsoft Access (2003) proved more time-consuming than predicted. Given the relatively high response rates achieved, the resulting shortage of time, and the fact that responses were fairly evenly spread across response groups, it was not deemed cost-effective to devote further time to following up non-respondents by phone.

**Reference**

1. Dillman, D.A. *Mail and Internet Surveys: The Tailored Design Method*; Wiley: Hoboken, NJ, USA, 2007.
