# Supplementary material for: Moles and Mole Control on British Farms, Amenities and Gardens after Strychnine Withdrawal
Source: Animals (Basel). 2016 Jun 8;6(6):39. doi: 10.3390/ani6060039 (PMC4929419; doi:10.3390/ani6060039)
Supplement: Supplementary file 1 [file animals-06-00039-s001.zip › Text S10.docx]

**Text S10: Factors Affecting Recent Mole Activity and Damage**

We examined factors affecting recent mole activity and damage. Where “recent mole activity” and “damage” are used here we refer respectively to mole activity in the previous year and perceived damage arising from this.

The proportion of respondents with historic mole presence that reported recent mole activity differed among respondent types (approximately 90% of farmers and amenities and only 46% of householders, Figure S3a). This suggests that farms and amenities with moles are more likely to experience regular mole activity, whereas mole activity may be more transient in gardens (which are generally smaller). The proportion of respondents with moles that reported recent mole activity also differed among regions, farm enterprises and amenity types, but not soil types. See Figures S3c,e,g), which suggest that respondents in the north of England and in Wales, livestock and mixed farms, and golf courses were most likely to observe regular mole activity on their land.

The proportion of respondents with recent mole activity that said this had caused damage differed among respondent types, regions, farm enterprise and amenity types. The groups most likely to do so were amenity managers, livestock farmers and respondents from the north of England and Wales (Figures S3b,d,h). There seems to be a link between respondents that reported regular mole activity and those that considered this to be damaging. Soil types were not related to the likelihood of respondents reporting regular mole activity, or those with recent activity considering it damage (Figures S3i,j).
