# Supplementary material for: Moles and Mole Control on British Farms, Amenities and Gardens after Strychnine Withdrawal
Source: Animals (Basel). 2016 Jun 8;6(6):39. doi: 10.3390/ani6060039 (PMC4929419; doi:10.3390/ani6060039)
Supplement: Supplementary file 1 [file animals-06-00039-s001.zip › animals-122455-suppl xml.docx]

Supplementary Materials: Moles and Mole Control on British Farms, Amenities and Gardens after Strychnine Withdrawal

Sandra E. Baker, Stephen A. Ellwood, Paul J. Johnson and David W. Macdonald

**Table S1.** Questionnaire responses for farmers, amenity managers and householders.

| **Questionnaire Survey Information** | **Farmers** | **Amenities** | **House-Holders** | **Total** |
| --- | --- | --- | --- | --- |
| Number of questionnaires sent | 1204 | 551 | 504 | 2259 |
| Number excluded from survey (undelivered/retired/deceased *etc*) | 61 | 25 | 23 | 109 |
| **Potential sample size after exclusions** | **1143** | **526** | **481** | **2150** |
| Number of responses | 720 | 303 | 242 | 1265 |
| **Response rate** | **63%** | **58%** | **50%** | **59%** |
| Number actively refusing to take part (blank returns *etc*) | 62 | 12 | 15 | 89 |
| **Percentage actively refusing to take part (blank returns *etc*)** | **5%** | **2%** | **3%** | **4%** |
| Number not responding at all | 361 | 211 | 224 | 796 |
| **Percentage not responding at all** | **32%** | **40%** | **47%** | **37%** |


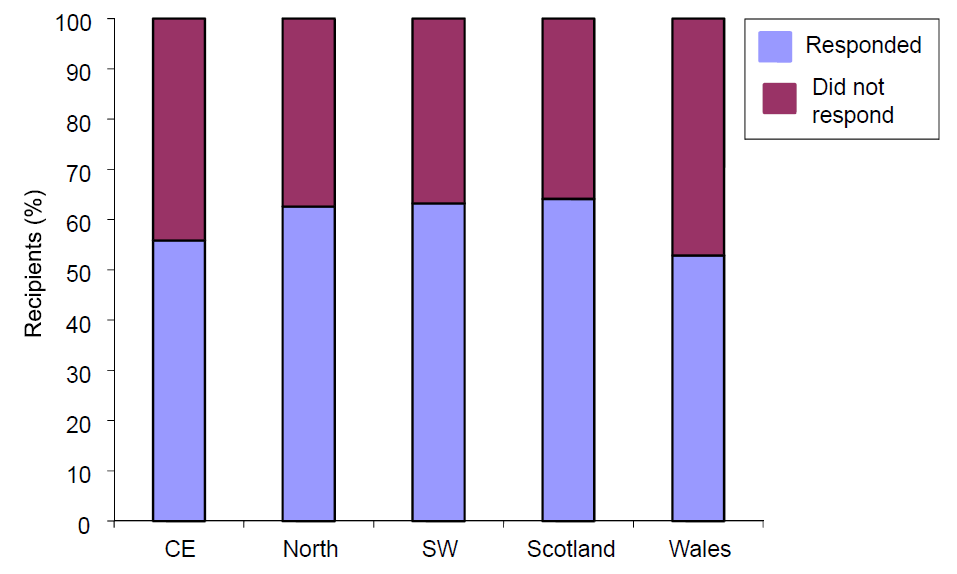


**Figure S1.** Response rates by region (CE = central and eastern England *n* = 1070, North = northern England *n* = 310, SW = south-western England *n* = 383, Scotland *n* = 259, Wales *n* = 123).


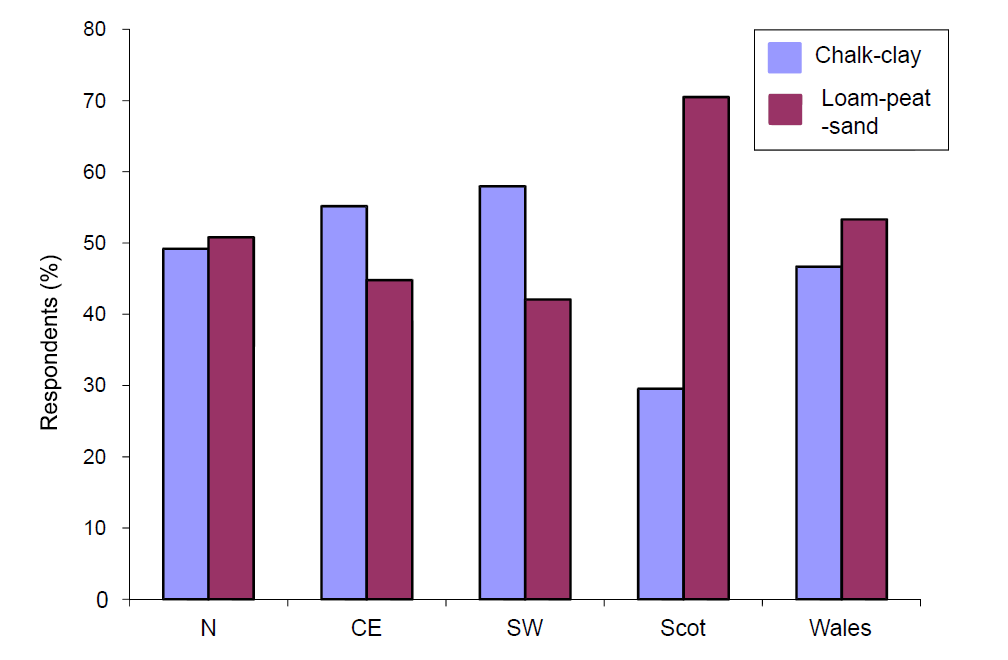


**Figure S2.** Soil type by region (N = northern England *n* = 124, CE = central and eastern England *n* = 433, SW = south-western England *n* = 202, Scot *n* = 122, Wales *n* = 45).

**
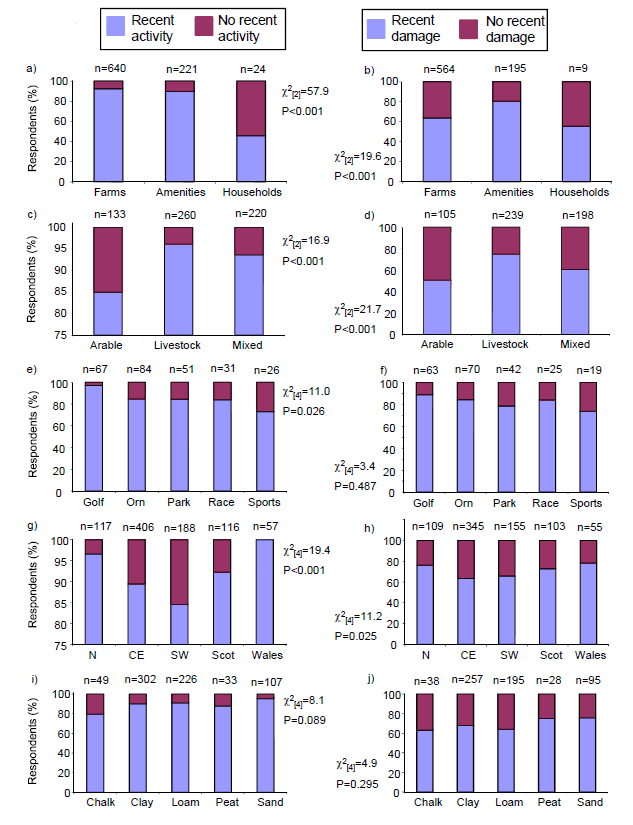
**

**Figure S3.** Recent mole activity and mole damage, respectively, by: (**a**, **b**) respondent type; (**c**, **d**) farm enterprise; (**e**, **f**) amenity type; (**g**, **h**) region; (**i**, **j**) soil type. Regions are: N = northern England, CE = central and eastern England, SW = south-western England, Scot = Scotland and Wales = Wales. Statistics shown are results of χ^2^ tests.


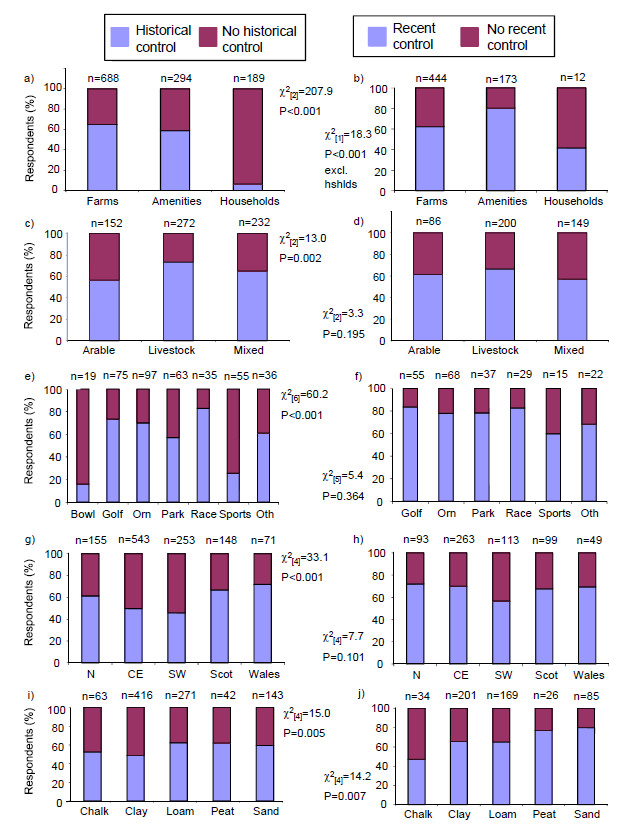


**Figure S4.** Historic and recent mole control by: (**a**, **b**) respondent type; (**c**, **d**) farm enterprise; (**e**, **f**) amenity type; (**g**, **h**) region; (**i**, **j**) soil type. Regions are: N = northern England, CE = central and eastern England, SW = south-western England, Scot = Scotland and Wales = Wales. Statistics shown are results of χ^2^ tests.

**
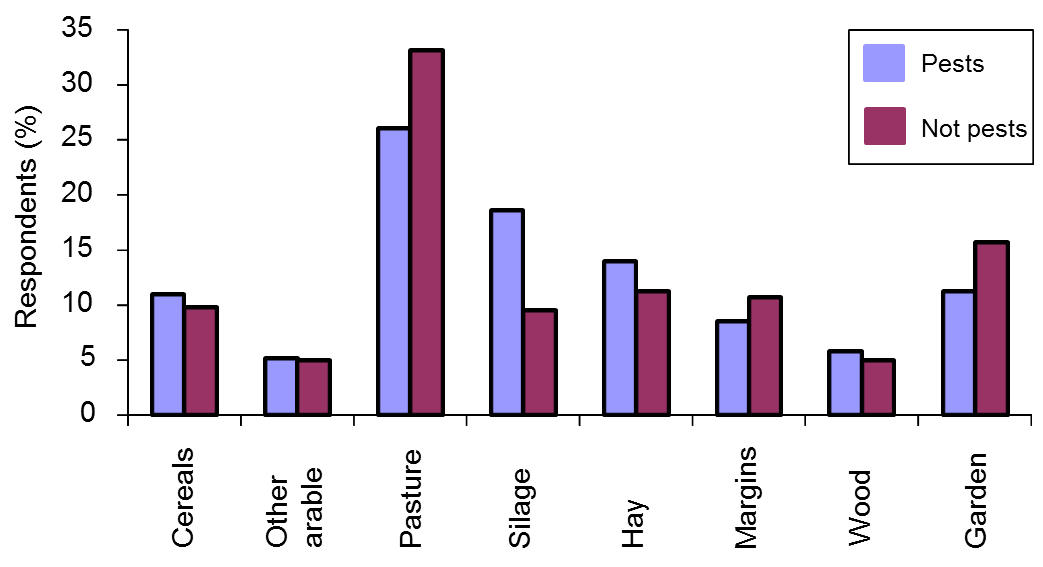
**

**Figure S5.** Habitats with reported mole activity in the previous year by farm mole pest status (sample sizes are: pest *n* = 1345, not pest *n* = 338).


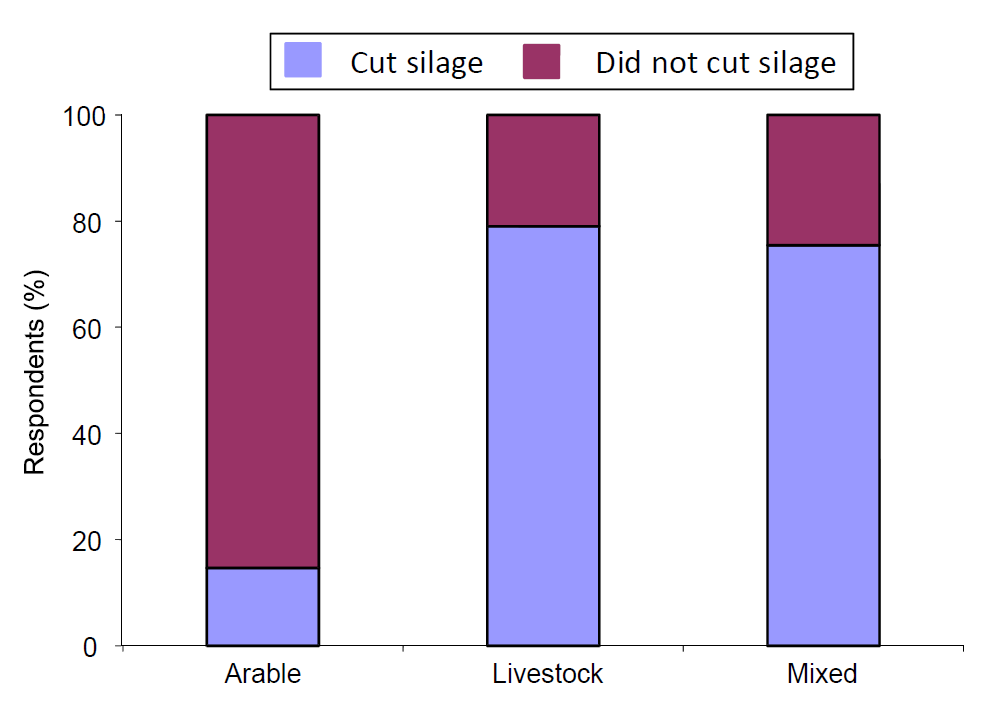


**Figure S6.** Silage production by farm enterprise type (arable *n* = 150, livestock *n* = 272, mixed *n* = 228).


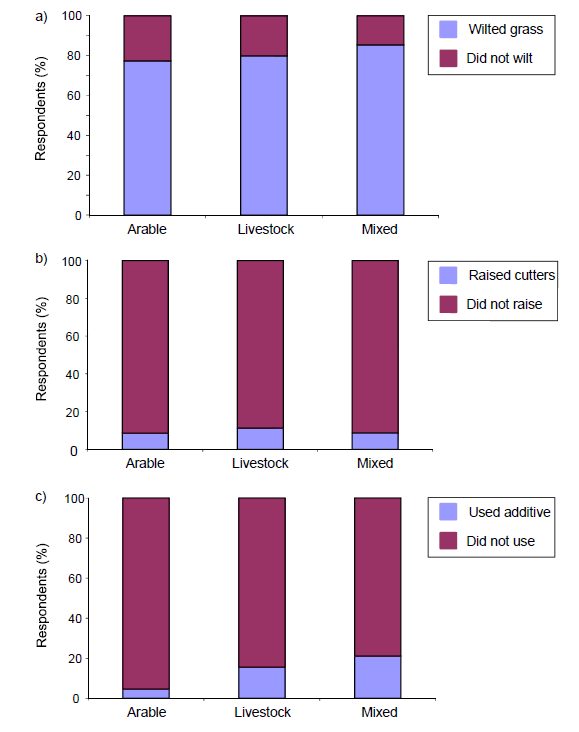


**Figure S7.** Silage damage mitigation measures taken by enterprise type: (**a**) wilting grass before baling (arable *n* = 22, livestock *n* = 213, mixed *n* = 171); (**b**) raising cutters (arable *n* = 23, livestock *n* = 213, mixed *n* = 171); and (**c**) using a silage additive (arable *n* = 22, livestock *n* = 213, mixed *n* = 171).


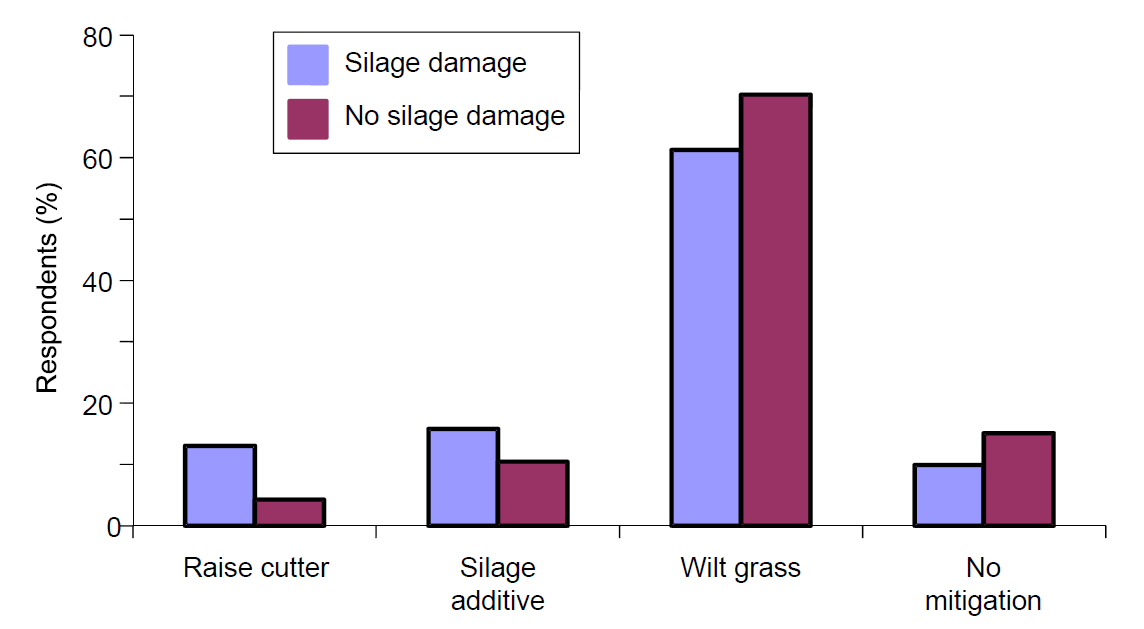


**Figure S8.** Reported silage damage by moles and silage protection measures taken by farmers (silage damage *n* = 253, no silage damage *n* = 212).


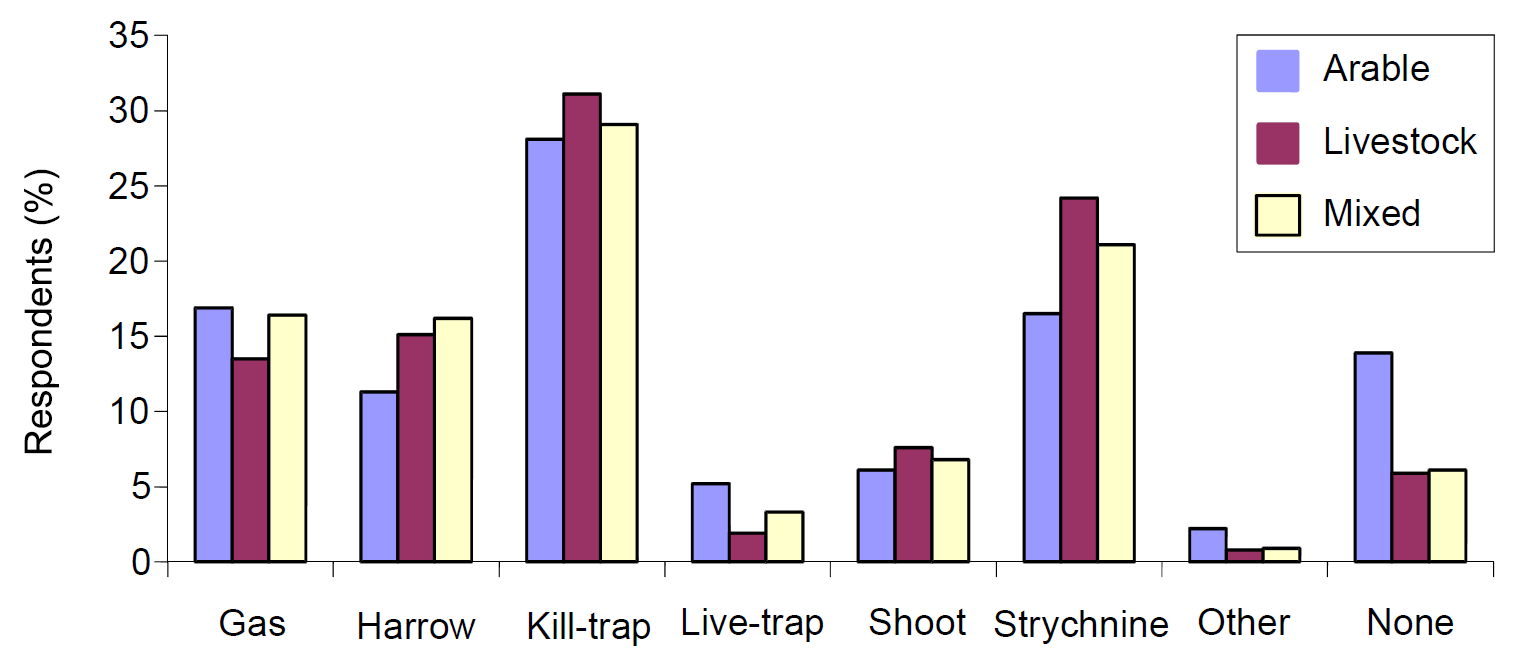


**Figure S9.** Proposed future control methods by farm enterprise type (sample sizes are: arable *n* = 231, livestock *n* = 476, mixed *n* = 426).


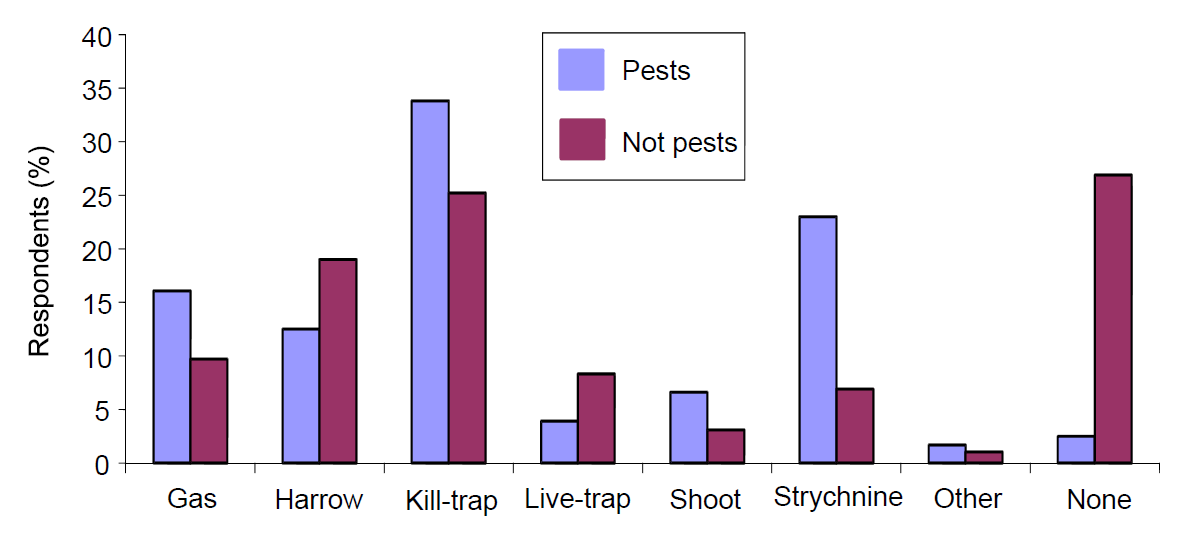


**Figure S10.** Proposed future control methods on farms and amenities by mole pest status (sample sizes are: pest *n* = 1181, not pest *n* = 290).


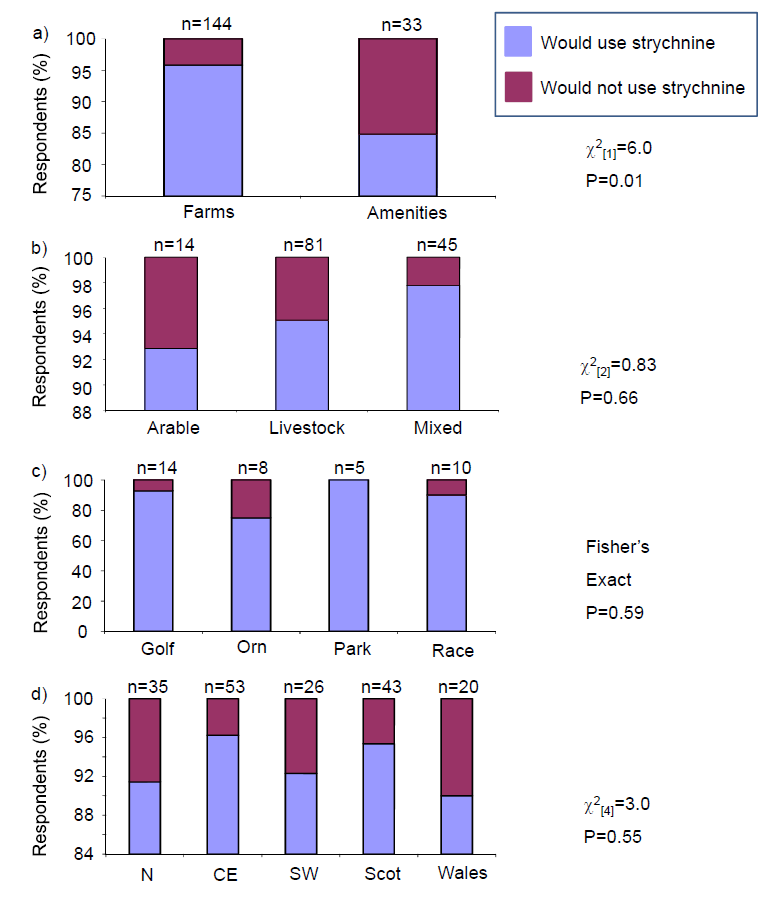


**Figure S11.** Intended future strychnine use by: (**a**) respondent type; (**b**) region; (**c**) farm enterprise;
(**d**) amenity type. Regions are: N = northern England, CE = central and eastern England, SW = south-western England, Scot = Scotland and Wales = Wales. Statistics shown are results of χ^2^ tests.


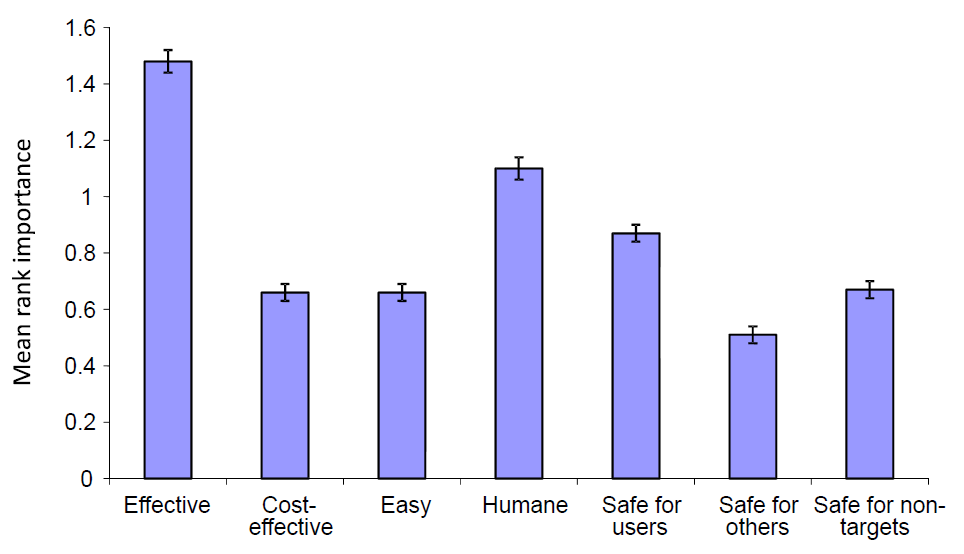


**Figure S12.** Important features of a mole control method based on mean ranked importance for all respondents (*n* = 1091).


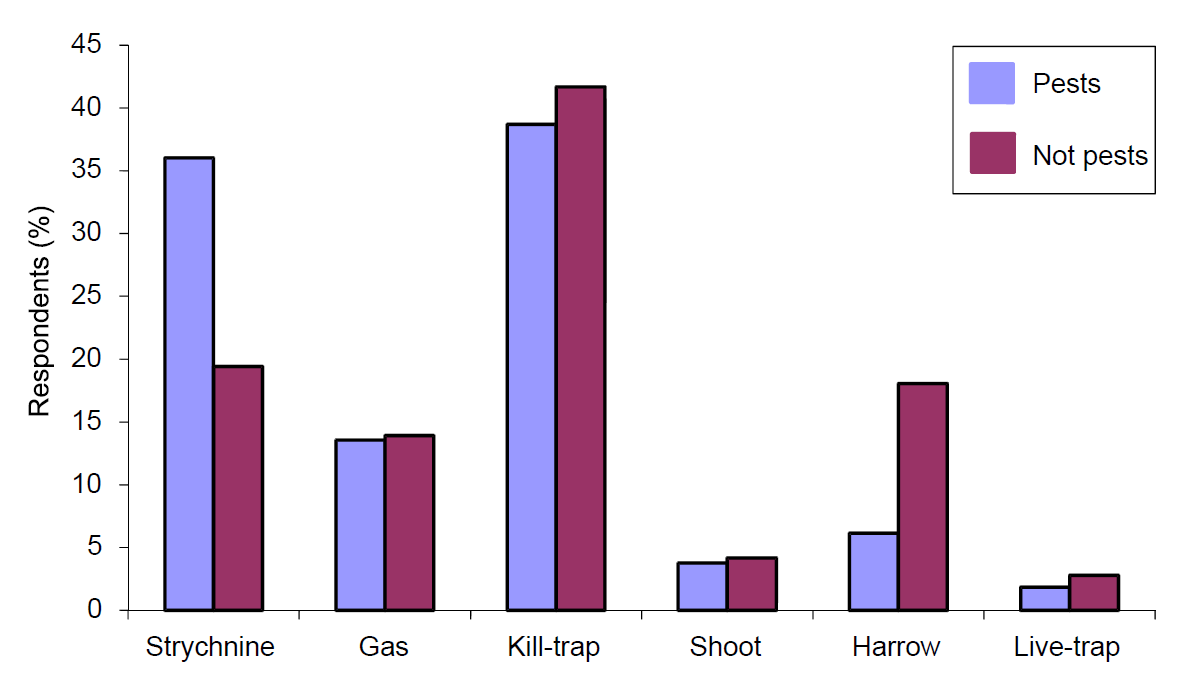


**Figure S13.** Respondent opinions of whether mole control methods were cost-effective by mole pest status (farmers and amenities only; pest *n* = 716, not pest *n* = 72).


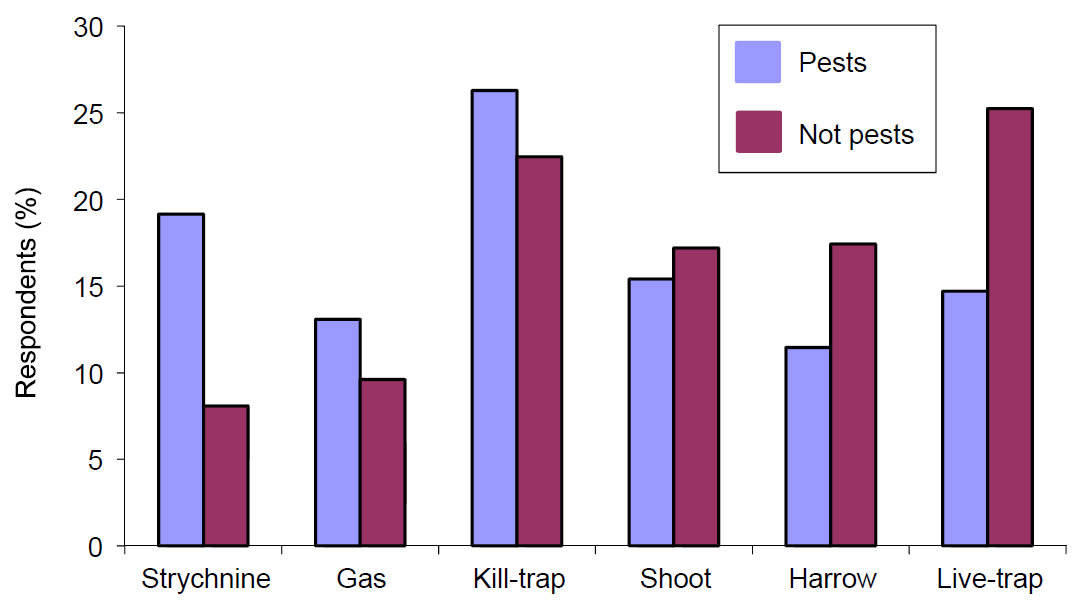


**Figure S14.** Respondent opinions of whether control methods were humane by mole pest status (farmers and amenities only; pest *n* = 1416, not pest *n* = 396).


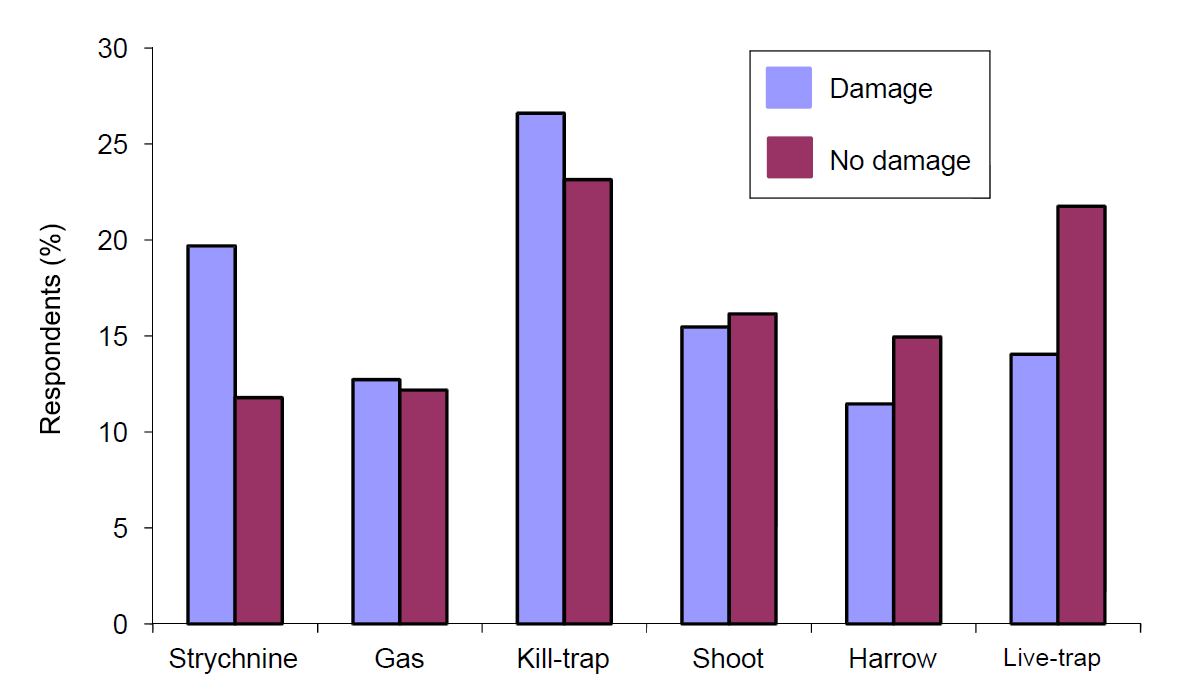


**Figure S15.** Respondent opinions of whether control methods were humane by mole damage status (farmers and amenities only; damage *n* = 1203; no damage *n* = 501).


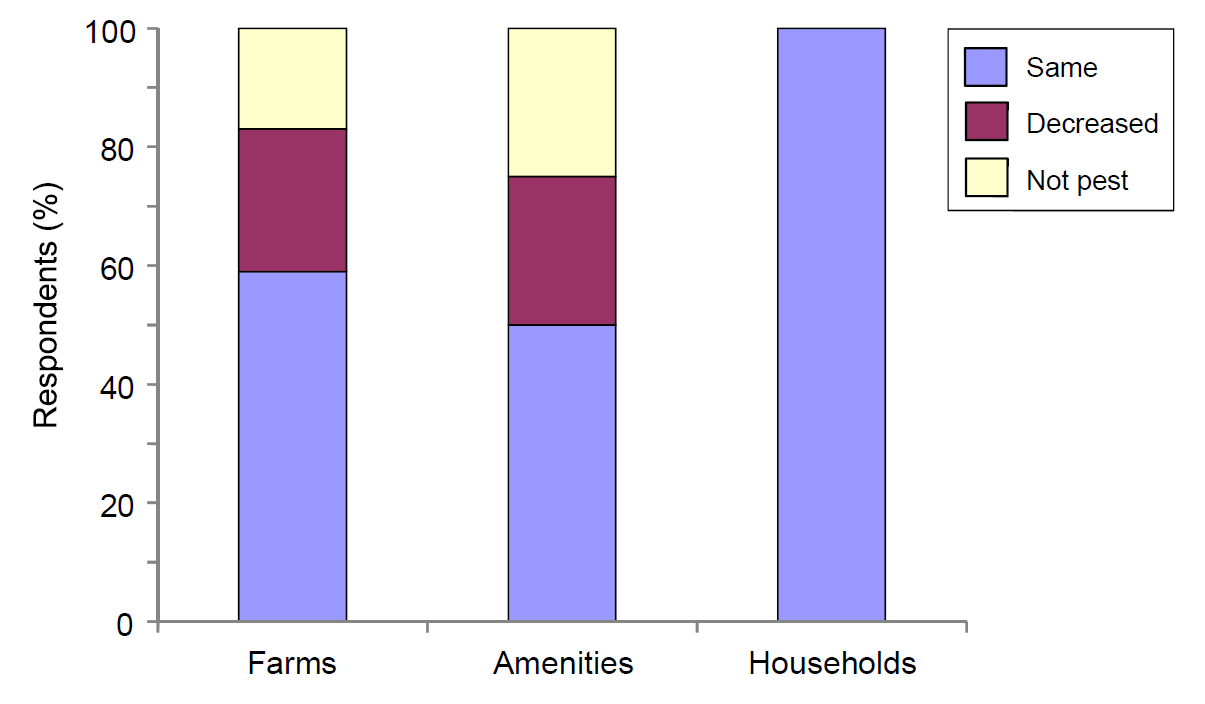


**Figure S16.** Ground-truthing participant opinions regarding recent changes in mole activity on their land over the previous five years (farms *n* = 17, amenities *n* = 8, households *n* = 4).

© 2016 by the authors; licensee MDPI, Basel, Switzerland. This article is an open access article distributed under the terms and conditions of the Creative Commons by Attribution (CC-BY) license (http://creativecommons.org/licenses/by/4.0/).
