# Supplementary material for: Moles and Mole Control on British Farms, Amenities and Gardens after Strychnine Withdrawal
Source: Animals (Basel). 2016 Jun 8;6(6):39. doi: 10.3390/ani6060039 (PMC4929419; doi:10.3390/ani6060039)
Supplement: Supplementary file 1 [file animals-06-00039-s001.zip › Text S1.docx]

**Text S1: Questionnaire Survey Method**

*The Tailored Design Method*

The Tailored Design Method for surveys aims to create respondent trust and perceptions of increased rewards and reduced costs for being a respondent that take into account features of the survey situation, and have as their goal the overall reduction of survey error. We employed various survey implementation measures to maximise response rates. These included: carefully worded multiple contacts with potential respondents ((a) pre-notice letter; (b) questionnaire and covering letter; (c) thank you/reminder postcard; (d) replacement questionnaire to non-responders and covering letter); real signatures on letters; real postage stamps on all correspondence; and stamped return envelopes [1]. We devised a contact schedule comprising four carefully worded postal contacts. Questionnaires were marked with individual identity numbers so that respondents could be excluded from future maillings. Questionnaires were produced as A4 double-sided booklets which have been shown to be more effective than other formats [1].

*Piloting*

The three questionnaires were piloted on a number of people including farmers, amenity managers and householders. During piloting we examined the way in which people interpreted questions (and, in multiple-choice questions, possible answers), whether they followed the directions of questions (e.g., “if no, go on to question X”), whether they considered the flow of questions to be logical, and how long they took to complete the questionnaire. Questionnaires were revised accordingly, and the revised versions then subjected to a second round of piloting and revision.

*Translation*

The questionnaires and contacts (letters and postcards) were translated into Welsh by a professional native Welsh translator so that recipients in Wales could be sent both English and Welsh versions.

*Example Contacts*

Example contacts (letters and questionnaire) are given in Text S2-S6 below.

*Recipient Database Abstraction*

We selected recipients for the survey from across England, Scotland and Wales in the following ways:

*Farmers (n=1204)*

We used the British towns list * randomly to select target towns and then entered target towns into www.thephonebook.bt.com, taking five farms listed under “Farmers” for each town. It was decided beforehand to take the first record on each of five pages (max five pages listed). If that entry looked inappropriate, e.g., a farm supplier or shop instead of a farm, then we selected the next on the list.

*Amenities (n=551)*

These consisted of the following:

Racecourses (*n* = 60): We included all 60 racecourses listed on The Racecourse Association’s website <http://www.britishracecourses.org/course_list.php>.

Bowls clubs (*n* = 30): We conducted a search for “lawn” and “crown green” listed clubs registered for Britain on <http://bowlsclub.org/search.php?>. Then we used randomly generated numbers ** to identify which pages of search results, and which entries on those pages, to select.

Golf courses (n=130): We referred to three websites:

<http://www.englishgolf-courses.co.uk/> (34 counties/county groups)

<http://www.welshgolfcourses.com/> (four regions)

<http://www.scotlands-golf-courses.com> (13 areas)

We used random numbers ** to select golf courses that represented the counties, regions, or areas according to relative frequency of courses in each.

Football clubs (*n* =130): We used the British towns list * to select towns and then entered these into [www.thephonebook.bt.com](http://www.thephonebook.bt.com), taking five clubs listed under “Football Grounds” for each town. It was decided beforehand to take the first record on each of five pages (max five pages listed). If that entry looked inappropriate, e.g., it was not a football club, we selected the next on the list.

Parks (*n* = 100): We used three methods of identifying parks. First we used <http://www.nationaltrust.org.uk/main/w-vh/w-visits/w-findaplace.htm> using their “parks” option for each of 30 counties (two per county) for England and Wales and http://www.nts.org.uk/
Property/Search/ to identify (approximately 10) gardens in Scotland. Then we searched for 20 Country Parks, and 20 municipal parks (mainly council-run) using [www.google.co.uk](http://www.google.co.uk). Then we deleted any duplicates (and any cross-over with gardens) and any excess (prioritising removal of private stately-home parks).

Ornamental gardens (*n* = 100): These were identified using the Royal Horticultural Society’s “Gardens to visit” list on <http://www.rhs.org.uk/rhsgardenfinder/gardenfinder.asp> (we took 38 gardens from the list of 39 that are apparently open free to RHS members—the other appeared to be transient (part of Chelsea Flower Show). Then we used <http://www.nationaltrust.org.uk/main/w-vh/w-visits/w-findaplace.htm> “gardens” option for each of 30 counties for England and Wales and <http://www.nts.org.uk/Property/Search/> to identify gardens in Scotland. Then we deleted any duplicates and excess entries.

*Householders (n = 504)*

We identified the most common 10 surnames in England and Wales for English and Welsh householders, and the most common 10 surnames in Scotland for Scottish householders. Names for England and Wales were taken from a list produced by the Office for National Statistics (<http://homepages.newnet.co.uk/dance/webpjd/offstats/names1-500.htm>) and those for Scotland from <http://www.gro-scotland.gov.uk>.Then we used a combination of towns selected randomly (page, row, position on row) from the British towns list *, and surnames (represented according to relative frequency in the population) to search for households using [www.thephonebook.bt.com](http://www.thephonebook.bt.com).

* The British towns list came from <http://www.information-britain.co.uk/towns.cfm>. Towns were selected using random numbers generated using the random number generator *. A certain number of towns was allocated to each of the 12 pages of towns, and then rows were selected from which to take the towns. For each new respondent type (e.g., householders, football clubs) it was decided beforehand whether to use the first, last or middle town from the row. NB towns from NI were passed over, and the next town used instead. If the [www.phonebook.bt.com](http://www.phonebook.bt.com) was being used in conjunction with the town list and the town did not appear on [www.phonebook.bt.com](http://www.phonebook.bt.com) then the next town was used.

** Random numbers were generated using <http://www.random.org/integers/>.

**Reference**

1. Dillman, D.A. *Mail and Internet Surveys: The Tailored Design Method*; Wiley: Hoboken, NJ, USA, 2007.
