# Supplementary material for: Moles and Mole Control on British Farms, Amenities and Gardens after Strychnine Withdrawal
Source: Animals (Basel). 2016 Jun 8;6(6):39. doi: 10.3390/ani6060039 (PMC4929419; doi:10.3390/ani6060039)
Supplement: Supplementary file 1 [file animals-06-00039-s001.zip › Text S2.doc]

21 June 2007

«Contact_name»

«Contact_position»,

«Address1»

«Address2»

«Address3»

«Address4»

We would like to invite you to take part in a new research project being conducted by the University of Oxford. In a few days time we will send you a request to fill out a brief questionnaire concerning farmers’ experience and views of mole activity and mole control on farms in Britain.

I am writing in advance because we have found that many people like to know beforehand that they will be contacted. The study is an important one that will help policy-makers to understand the extent of mole activity, whether there is a need for mole control, and if so whether appropriate control methods are available. This information will help to direct future research in these areas.

We would greatly appreciate you taking a few minutes to complete and return the questionnaire. Only with the generous help of people like you can our research be successful.

Thank you for your time and consideration.

Yours sincerely

Dr Sandra Baker

Wildlife Research Scientist

Wildlife Conservation Research Unit

University of Oxford
