# Supplementary material for: Moles and Mole Control on British Farms, Amenities and Gardens after Strychnine Withdrawal
Source: Animals (Basel). 2016 Jun 8;6(6):39. doi: 10.3390/ani6060039 (PMC4929419; doi:10.3390/ani6060039)
Supplement: Supplementary file 1 [file animals-06-00039-s001.zip › Text S3.doc]

28 June 2007

«Contact_name»

«Contact_position»,

«Address1»

«Address2»

«Address3»

«Address4»

I am writing to ask your help in a study of moles on farms, being conducted by the University of Oxford. The goal of this study is to learn about mole activity and mole control across Britain.

It is my understanding that you are currently farming. We are contacting a sample of farmers from all regions in England, Wales and Scotland to ask about their experiences and views of mole activity and control.

The survey will generate unique information about moles in Britain including the scale of mole activity on farms, whether or not this causes inconvenience to farmers, whether farmers control moles, and farmers’ opinions about the control methods available. **By completing the questionnaire you will contribute a valuable insight into mole management in Britain. It is equally important that you take part, whether or not you have moles on your land, and regardless of your views about them.** Results from this survey will be summarised and provided to policy-makers, together with proposals for how the results might be used to help farmers in future.

The short questionnaire is self-explanatory and should only take about 10-15 minutes to complete. Your answers are completely confidential and will be released only as summaries in which no individual’s answers can be identified. Information from the study will be analysed at the University of Oxford and will not be made available to third parties. If for some reason you prefer not to respond, please let us know by returning the blank questionnaire in the enclosed stamped envelope.

You will be able to view a summary of the results on our website when these are available ([www.wildcru.org/molesurvey](http://www.wildcru.org/molesurvey)). If you have any questions or comments about this study, I would be happy to talk with you. You can contact me on 01865 393114, at [molesurvey@zoo.ox.ac.uk](mailto:molesurvey@zoo.ox.ac.uk), or at the above address.

Thank you very much for helping with this important study.

Yours sincerely

Dr Sandra Baker (Wildlife Research Scientist)

Wildlife Conservation Research Unit, University of Oxford	F0001

**P.S. If by some chance we have made a mistake and you are not farming, please write a note about this on the back of the questionnaire and return the rest of it blank. Many thanks.**

This survey is endorsed by the following organisations: National Farmers’ Union of Wales, The National Farmers’ Union of Scotland, The British Association for Shooting and Conservation, The Game Conservancy Trust, The National Trust, The Royal Horticultural Society, The Racecourse Association, The Airport Operators’ Association, The British and International Golf Greenkeepers’ Association.
