# Supplementary material for: Moles and Mole Control on British Farms, Amenities and Gardens after Strychnine Withdrawal
Source: Animals (Basel). 2016 Jun 8;6(6):39. doi: 10.3390/ani6060039 (PMC4929419; doi:10.3390/ani6060039)
Supplement: Supplementary file 1 [file animals-06-00039-s001.zip › Text S4.doc]

**Please help find better ways of managing moles on farms**

***Oxford University Mole Survey 2007***

**START HERE**

**Please answer all questions unless directed to move on to another**

**Your farm**

**(1) What is your main farming enterprise?**

Arable	

Livestock	

Mixed	

Other	

(Please specify	)

**(2) What is your position on the farm?**

Owner or tenant farmer	

Farm manager (staff)	

Other farm staff	

Other	

(Please specify)	)

**(3) How long have you been at this farm?**

years

**(4) What is the main soil type on your farm? *(Mark one only).***

Clay	

Chalky	

Loamy	

Peaty	

Sandy	

Silty	

Don’t know	

**(5) What is the size of your farm?**

hectares OR	acres

**(6) *Roughly* what area of your farm was occupied by each of the following over the past year (June 2006-May 2007)?**

Cereals	ha OR	acres

Other arable	ha OR	acres

Grass cut for silage	ha OR	acres

Grass cut for hay	ha OR	acres

Pasture	ha OR	acres

Field margins	ha OR	acres

Woodland	ha OR	acres

Lawns / gardens	ha OR	acres

Water bodies	ha OR	acres

Other	ha OR	acres

(Please specify	)

1

**(7) Did you cut silage over the past year?**

Yes	

No		If no, go to question 9

**(8) If yes, which of the following did you do? *(Mark all that apply).***

Raise the cutters to avoid molehills	

Wilt grass after cutting	

Use a silage additive	

None of the above	

**(9) Which county (England / Wales) or district (Scotland) is your farm in?**

county / district

**(10) What is the first half of your farm postcode, e.g., OX13?**

first half of postcode

**Moles on your farm**

**(11) Have you ever had moles on your farm?**

Yes	

No		If no, or don’t know,

Don’t know		go to question 20

**(12) Do you consider moles on your farm to be pests, or not to be pests?**

Pest	

Not a pest	

Don’t know	

**(13) Were molehills or mole tunnels present on your farm over the past year (June 2006–May 2007)?**

Yes	

No		If no, go to question 20

**(14) If yes, which of the following habitats did they occur on over the past year? *(Mark all that apply).***

Cereals	

Other arable	

Grass cut for silage	

Grass cut for hay	

Pasture	

Field margins	

Woodland	

Lawns / gardens	

Other	

(Please specify)	)

**(15) Would you say that mole activity on your farm was zero, low or high (compared to other seasons) in each season over the past year? *(Mark one box per season).***

**Don’t**

***Z*ero Low High know**

Summer (Jun-Aug 2006)				

Autumn (Sep-Nov 2006)				

Winter (Dec 2006-Feb 2007)				

Spring (Mar-May 2007)				

**(16) Please rank the three months over the past year (June 2006-May 2007) in which mole activity was greatest on your farm. *(Write the name of the month with the greatest activity next to ‘Rank 1’, and so on until you have ranked the top three months).***

Rank 1	month

Rank 2	month

Rank 3	month

**(17) Would you say that the mole activity on your farm over the past year caused any damage?**

Yes	

No		If no, or don’t know,

Don’t know		go to question 20

**(18) If yes, what sort of damage did moles cause? *(Mark all that apply).***

Silage spoiled	

Pasture covered	

Animals injured	

Machinery damaged	

Plants damaged	

Watercourses / drainage damaged	

Weed invasion on molehills	

Other	

Please specify)	)

**(19) If you know how much money this damage was worth (excluding efforts to control moles) please say how much below. If you don’t know, please mark ‘don’t know’.**

Cost of damage in past year: £

Don’t know	

**Mole control on your farm**

**(20) Has anybody ever used mole control on your farm during your time there? *(Please answer even if you reported no mole activity in the past year).***

Yes	

No		If no, go to question 30

**(21) If yes, which methods were used for controlling moles? *(Mark all that apply).***

Strychnine poison	

Kill-trapping	

Gassing (phosphine)	

Shooting	

Harrowing or rolling hills / tunnels	

Live-trapping and release	

Other	

(Please specify	)

2

**(22) Do you consider the following methods to be effective (Eff) or not effective (Not) at controlling moles on farms?**

***(Mark one box per method).*	Don’t**

**Eff	Not know**

Strychnine poison			

Kill-trapping			

Gassing (phosphine)			

Shooting			

Harrowing or rolling hills / tunnels			

Live-trapping and release			

**(23) Do you consider the following methods to be cost-effective (C-E) or not cost-effective (Not) at controlling moles on farms?**

***(Mark one box per method).*	Don’t**

**C-E	Not know**

Strychnine poison			

Kill-trapping			

Gassing (phosphine)			

Shooting			

Harrowing or rolling hills / tunnels			

Live-trapping and release			

**(24) Over the past year (June 2006-May 2007), did anybody use mole control on your farm? *(Please answer even if you reported no mole activity).***

Yes	

No		If no, go to question 30

**(25) If yes, which methods were used for controlling moles over the past year?**

***(Mark all that apply).***

Strychnine poison	

Kill-trapping	

Gassing (phosphine)	

Shooting	

Harrowing or rolling hills / tunnels	

Live-trapping and release	

Other	

(Please specify	)

**(26) In which months was mole control conducted on your farm? *(Mark all that apply)*.**

June 2006	

July 2006	

Aug 2006	

Sept 2006	

Oct 2006	

Nov 2006	

Dec 2006	

Jan 2007	

Feb 2007	

Mar 2007	

Apr 2007	

May 2007	

Don’t know	

**27) Who carried out mole control over the past year? *(Mark all that apply).***

You / your family / farm staff	

Professional pest controller	

Other	

(Please specify	)

**28) *Roughly* how many man-hours were spent on mole control on your farm over the past year by the following people?**

You / your family / farm staff	hours

Professional pest controller	hours

Other	hours

(Please specify	)

Don’t know	

**29) *Roughly* how much money was spent on mole control on your farm over the past year?**

Professional pest control	£

Materials for own / staff use	£

Don’t know	

**Your opinions about mole control**

**30) Do you consider the following mole control methods to be humane (Hum) or not humane (Not)?**

***(Mark one box per method).*	Don’t**

**Hum Not know**

Strychnine poison			

Kill-trapping			

Gassing (phosphine)			

Shooting			

Harrowing or rolling hills / tunnels			

Live-trapping and release			

**31) If moles were present on your land in future, which methods of mole control would you consider using? *(Mark all that apply).***

I would not control moles at all	

Strychnine poison (if legally available)	

Kill-trapping	

Gassing (phosphine)	

Shooting	

Harrowing or rolling hills / tunnels	

Live-trapping and release	

Other	

(Please specify	)

**32) Please rank the three features of a mole control method that are most important to you. *(Give a 1 to the most important, and so on until you have ranked the top three).***

Cost-effective	Rank

Easy to use	Rank

Effective	Rank

Humane	Rank

Safe to users	Rank

Safe to other people	Rank

Safe to non-target animals	Rank

3

**Strychnine poison**

**33) Has strychnine been used to control moles on your farm in the last 5 years (or during your time there if less than 5 years)?**

Yes	

No		If no, go to question 38

**34) If yes, who used strychnine on your farm? *(Mark all that apply).***

You / your family / farm staff	

Professional pest controller	

Other	

(Please specify	)

**35) Which single main method of mole control has been used on your farm since strychnine was banned in September 2006? *(Mark one only).***

Moles have not been controlled at all	

Kill-trapping	

Gassing (phosphine)	

Shooting	

Harrowing or rolling hills / tunnels	

Live-trapping and release	

Other	

(Please specify	)

**36) Was this method more effective, about as effective, or less effective than strychnine?**

More effective than strychnine	

About as effective as strychnine	

Less effective than strychnine	

Don’t know	

**37) If strychnine became legally available again, for mole control, would it be used on your farm?**

Yes	

No	

Don’t know	

**Follow-up study**

**38) Would you be interested in taking part in a small follow-up study on mole activity and control? This would involve a member of our team visiting your farm to ask a few more questions and assess mole activity levels. If so, please write your contact details below (without obligation).**

Your name

Address

Town / postcode

Phone number

**THANK YOU FOR COMPLETING THIS SURVEY. PLEASE RETURN YOUR QUESTIONNAIRE TO ME IN THE STAMPED ENVELOPE PROVIDED.**

**Thank you for taking the time to complete this questionnaire. Your assistance in providing this information is very much appreciated. If there is anything else you would like to tell us about this survey, or about mole activity and mole control on your farm, please do so in the space provided below.**

This survey is endorsed by the following organisations:

National Farmers’ Union of Wales, The National Farmers’ Union of Scotland, The British Association for Shooting and Conservation, The Game Conservancy Trust, The National Trust, The Royal Horticultural Society, The Racecourse Association, The Airport Operators’ Association, The British and International Golf Greenkeepers’ Association.

Please return your completed questionnaire in the stamped envelope provided to:

Dr Sandra Baker

The Wildlife Conservation Research Unit,

University of Oxford,

Tubney House, Abingdon Road,

Tubney, Abingdon,

Oxfordshire OX13 5QL

F0001

4
