# Supplementary material for: Moles and Mole Control on British Farms, Amenities and Gardens after Strychnine Withdrawal
Source: Animals (Basel). 2016 Jun 8;6(6):39. doi: 10.3390/ani6060039 (PMC4929419; doi:10.3390/ani6060039)
Supplement: Supplementary file 1 [file animals-06-00039-s001.zip › Text S5.doc]

| **Oxford University Mole Survey 2007**  Last week a questionnaire seeking your opinion about mole activity and mole control was sent to you. Your name was drawn randomly from a list of farmers in your area.  If you have already completed and returned the questionnaire to us, please accept our sincere thanks. If not, please do so today. We are especially grateful for your help because it is only by asking people like you to share your experiences that we can understand the extent of mole activity, whether there is a need for mole control, and if so whether appropriate control methods are available.  If you didn’t get a copy of the questionnaire, or if you can’t find it, please contact us on (01865) 393114, or [molesurvey@zoo.ox.ac.uk](mailto:molesurvey@zoo.ox.ac.uk) and we will get another one in the post to you today.  Yours sincerely  Dr Sandra Baker, Wildlife Research Scientist  Wildlife Conservation Research Unit, University of Oxford |  | **Oxford University Mole Survey 2007**  Last week a questionnaire seeking your opinion about mole activity and mole control was sent to you. Your name was drawn randomly from a list of farmers in your area.  If you have already completed and returned the questionnaire to us, please accept our sincere thanks. If not, please do so today. We are especially grateful for your help because it is only by asking people like you to share your experiences that we can understand the extent of mole activity, whether there is a need for mole control, and if so whether appropriate control methods are available.  If you didn’t get a copy of the questionnaire, or if you can’t find it, please contact us on (01865) 393114, or [molesurvey@zoo.ox.ac.uk](mailto:molesurvey@zoo.ox.ac.uk) and we will get another one in the post to you today.  Yours sincerely  Dr Sandra Baker, Wildlife Research Scientist  Wildlife Conservation Research Unit, University of Oxford |
| --- | --- | --- |
|  |  |  |
| **Oxford University Mole Survey 2007**  Last week a questionnaire seeking your opinion about mole activity and mole control was sent to you. Your name was drawn randomly from a list of farmers in your area.  If you have already completed and returned the questionnaire to us, please accept our sincere thanks. If not, please do so today. We are especially grateful for your help because it is only by asking people like you to share your experiences that we can understand the extent of mole activity, whether there is a need for mole control, and if so whether appropriate control methods are available.  If you didn’t get a copy of the questionnaire, or if you can’t find it, please contact us on (01865) 393114, or [molesurvey@zoo.ox.ac.uk](mailto:molesurvey@zoo.ox.ac.uk) and we will get another one in the post to you today.  Yours sincerely  Dr Sandra Baker, Wildlife Research Scientist  Wildlife Conservation Research Unit, University of Oxford |  | **Oxford University Mole Survey 2007**  Last week a questionnaire seeking your opinion about mole activity and mole control was sent to you. Your name was drawn randomly from a list of farmers in your area.  If you have already completed and returned the questionnaire to us, please accept our sincere thanks. If not, please do so today. We are especially grateful for your help because it is only by asking people like you to share your experiences that we can understand the extent of mole activity, whether there is a need for mole control, and if so whether appropriate control methods are available.  If you didn’t get a copy of the questionnaire, or if you can’t find it, please contact us on (01865) 393114, or [molesurvey@zoo.ox.ac.uk](mailto:molesurvey@zoo.ox.ac.uk) and we will get another one in the post to you today.  Yours sincerely  Dr Sandra Baker, Wildlife Research Scientist  Wildlife Conservation Research Unit, University of Oxford |
