# Supplementary material for: Moles and Mole Control on British Farms, Amenities and Gardens after Strychnine Withdrawal
Source: Animals (Basel). 2016 Jun 8;6(6):39. doi: 10.3390/ani6060039 (PMC4929419; doi:10.3390/ani6060039)
Supplement: Supplementary file 1 [file animals-06-00039-s001.zip › Text S6.doc]

**WILDLIFE CONSERVATION RESEARCH UNIT** University of Oxford

Director: Professor David W. Macdonald MA DPhil DSc

*Tubney House*

*Abingdon Road*

*Tubney*

*Abingdon OX13 5QL*

*Telephone: (01865) 393100*

*Fax: (01865) 393101*

*Email (general enquiries):*

*wcru@zoology.oxford.ac.uk*

18 July 2007

«Contact_name»

«Contact_position»,

«Address1»

«Address2»

«Address3»

«Address4»

About three weeks ago I sent a questionnaire to you that asked about your experiences of mole activity and mole control.

The comments of people who have already responded include a wide variety of opinions about mole activity and mole control. Many have described their experiences, both good and bad, of moles on their land. We think the results are going to be very useful to policy-makers, and ultimately people like you.

We are writing again because of the importance that your questionnaire has for helping to get accurate results. Although we have sent questionnaires to people living in every region in Britain, it is only by hearing from nearly everyone in the sample that we can be sure that the results are truly representative.

**A few people have written to say that they should not have received the questionnaire because they are no longer farming. If this concern applies to you, please let us know on the cover of the questionnaire and return it in the enclosed envelope so that we can delete your name from the mailing list.**

A comment on our survey procedures. A questionnaire identification number is printed on the back cover of the questionnaire so that we can check your name off the mailing list when it is returned. Your name will not be connected to your answers unless you choose to give us your details for the follow-up study. Protecting the confidentiality of people’s answers is very important to us, as well as the University.

**We hope that you will fill out and return the questionnaire soon, but if for any reason you prefer not to answer it, please let us know by returning a note or blank questionnaire in the enclosed stamped envelope.**

Yours sincerely

Dr Sandra Baker (Wildlife Research Scientist)	**(Tel 01865 393114)**

Wildlife Conservation Research Unit, University of Oxford	F0001

This survey is endorsed by the following organisations: National Farmers’ Union of Wales, The National Farmers’ Union of Scotland, The British Association for Shooting and Conservation, The Game Conservancy Trust, The National Trust, The Royal Horticultural Society, The Racecourse Association, The Airport Operators’ Association, The British and International Golf Greenkeepers’ Association.
